# A RNA-Seq Analysis of the Rat Supraoptic Nucleus Transcriptome: Effects of Salt Loading on Gene Expression

**DOI:** 10.1371/journal.pone.0124523

**Published:** 2015-04-21

**Authors:** Kory R. Johnson, C. C. T. Hindmarch, Yasmmyn D. Salinas, YiJun Shi, Michael Greenwood, See Ziau Hoe, David Murphy, Harold Gainer

**Affiliations:** 1 Bioinformatics Section, Information Technology and Bioinformatics Program, National Institute of Neurological Disorders and Stroke, National Institutes of Health, Bethesda, MD, 20892, United States of America; 2 Laboratory of Neurochemistry, National Institute of Neurological Disorders and Stroke, National Institutes of Health, Bethesda, MD, 20892, United States of America; 3 School of Clinical Sciences, Dorothy Hodgkin Building, University of Bristol, Bristol, England, BS1 3NY; 4 Department of Physiology, Faculty of Medicine, University of Malaya, Kuala Lumpur, Malaysia, 50603; National Institutes of Health, UNITED STATES

## Abstract

Magnocellular neurons (MCNs) in the hypothalamo-neurohypophysial system (HNS) are highly specialized to release large amounts of arginine vasopressin (Avp) or oxytocin (Oxt) into the blood stream and play critical roles in the regulation of body fluid homeostasis. The MCNs are osmosensory neurons and are excited by exposure to hypertonic solutions and inhibited by hypotonic solutions. The MCNs respond to systemic hypertonic and hypotonic stimulation with large changes in the expression of their Avp and Oxt genes, and microarray studies have shown that these osmotic perturbations also cause large changes in global gene expression in the HNS. In this paper, we examine gene expression in the rat supraoptic nucleus (SON) under normosmotic and chronic salt-loading SL) conditions by the first time using “new-generation”, RNA sequencing (RNA-Seq) methods. We reliably detect 9,709 genes as present in the SON by RNA-Seq, and 552 of these genes were changed in expression as a result of chronic SL. These genes reflect diverse functions, and 42 of these are involved in either transcriptional or translational processes. In addition, we compare the SON transcriptomes resolved by RNA-Seq methods with the SON transcriptomes determined by Affymetrix microarray methods in rats under the same osmotic conditions, and find that there are 6,466 genes present in the SON that are represented in both data sets, although 1,040 of the expressed genes were found only in the microarray data, and 2,762 of the expressed genes are selectively found in the RNA-Seq data and not the microarray data. These data provide the research community a comprehensive view of the transcriptome in the SON under normosmotic conditions and the changes in specific gene expression evoked by salt loading.

## Introduction

The hypothalamo-neurohypophysial system (HNS) is the major neuroendocrine system through which the brain maintains body fluid homeostasis [1]. The HNS is composed of two large neuronal cell types, the vasopressin (Avp) and oxytocin (Oxt) peptide synthesizing magnocellular neurons (MCNs) that are located in discrete nuclei, the supraoptic (SON) and paraventricular (PVN) nuclei in the hypothalamus [2,3]. These cells are highly specialized to release large amounts of Avp or Oxt into the blood stream and to play crucial roles in the regulation of salt and water balance, lactation, and affiliative behavior [4,5,6]. The high rates of neuropeptide synthesis, transport, and release in Avp and Oxt MCNs have made these cells important experimental models for the study of peptidergic neuronal cell biology [4,7].

The MCNs respond to perturbations in systemic water balance by releasing large amounts of stored Avp and Oxt into the general circulation. The MCNs accomplish this, in part, by being osmosensory neurons that are intrinsically osmosensitive and are excited by hypertonic stimulation which produce decreases in cell volume and subsequent activation of non-selective cation channel conductances [8,9] and are inhibited by membrane stretch due to increases in their cell volumes in hypotonic solutions [8]. The MCNs also respond to systemic hypertonic and hypotonic stimulation with large changes in the expression of their Avp and Oxt genes [7]. There are established experimental paradigms to produce such osmotic stimulation in vivo. Plasma osmolality can be increased acutely (by the intraperitoneal injection of 1.5 M NaCl), or chronically, either by complete fluid deprivation for up to 3 days (referred to as “dehydration”) or by replacement of the normal drinking diet with 2% (wt/vol) NaCl for 5–7 days (referred to as “salt-loading”). Chronic dehydration or salt-loading also produces large increases in steady state volumes of the MCNs, including ultrastructural changes that suggest global increases in transcription and translation in these neurons under these conditions [10,11,12,13,14,15]. Several laboratories have studied changes in the HNS transcriptome using microarray analyses under normosmotic conditions and after chronic osmotic perturbation and have demonstrated that there are substantial global changes in gene expression in the HNS during chronic, systemic hyperosmolarity [16,17,18,19] and hypoosmolarity [20,21].

In this paper, we examine gene expression in the rat SON under normosmotic (euhydrated) and chronic salt-loading conditions for the first time using “new-generation”, RNA sequencing (RNA-Seq) methods. We focus on the SON in the HNS because this anatomically defined nucleus can be cleanly and easily dissected from the rat hypothalamus, and is a relatively simple neuroanatomic site, in that it contains only two neuronal phenotypes, the Oxt and Avp MCNs [2]. In addition, we compare SON transcriptomes determined by RNA-Seq methods with SON transcriptomes determined by Affymetrix microarray methods in rats under the same osmotic conditions. These data provide the research community a comprehensive view of the transcriptome in the SON under normosmotic conditions and after changes in specific gene expression evoked by salt loading.

## Methods

### Animals

For the RNA-Seq studies adult male Sprague-Dawley rats (270 g–370 g) obtained from Charles River Laboratories (Wilmington, MA) were maintained under normal laboratory conditions (temperature: 21–23°C, 12 h light-dark cycles with light on at 6:00 AM) with access to unlimited food and drinking water. All procedures were carried out in accordance with the National Institutes of Health (NIH) guidelines on the care and use of animals and an animal study protocol No. 1278 approved by the National Institute of Neurological Disorders and Stroke (NINDS) Animal Care and Use Committee.

For the microarray and qRT-PCR experiments all procedures were approved by the University of Bristol Ethical Review Committee and were carried out in accordance with the Animals (Scientific Procedures) Act, 1986. Adult male Sprague-Dawley rats (10–12 weeks old; Harlan Sera-lab, Loughborough, UK) were maintained in standardized temperature (22±1°C), humidity (50±5%) and diurnal conditions (10 hours light, 14 hours dark; lights on at 0700). All animals were allowed to acclimatize for 5 days prior to the start of the experiment at which point animals (n = 5) were given 2% saline (w/v) for 5 days. Control animals had normal access to drinking water and all animals had access to regular chow ad libitum. Sacrifice time was always between 1100 and 1300 each day.

### Chronic Salt loading

To induce chronic hyperosmolality (hypernatremia), rats were given 2% NaCl solution ad libitum as their only drinking fluid for 5 d. Euhydrated (Control) rats had free access to drinking water over the same time period. Daily fluid intake was monitored. Rats were weighed, euthanized by decapitation and their brains removed and quickly frozen on dry ice (see below). The control rats were killed at the same time as the salt loaded rats. Rats tolerate salt loading very well and no special care is necessary. This method is standard in the field to produce systemic hyperosmolarity & hypernatremia. Some papers describing the use this method have previously been published [15,22,23,24,25,26,27]. One of the consequences of chronic salt loading is a nearly two-fold increase in cell volume of the MCNs [15], confirmed in this study) which is known to be accompanied by a global increase in gene expression in the MCNs [7]. Note also the doubling of the total

### Tissue Preparation for Laser Capture Microdissection (LCM)

The rats were euthanized by decapitation, their brains were quickly removed from the skull, and the cerebellum and brain stem were removed and discarded. The frozen brain was then trimmed at the level of the anterior commissure prior to sectioning. The brain block containing the hypothalamus was then rapidly frozen on dry ice and stored at −80°C until sectioning was done. Details about this procedure were previously published [28,29]. Briefly, the frozen brain was mounted onto Tissue-Tek OCT compound (Sakura Finetek USA, Torrance, CA) on a chuck with the caudal portion of the brain placed down on the chuck, and the brain on the chuck was placed in the cryostat (Reichert-Jung 2800, Frigocut, Heidelberg, Germany) for twenty minutes to equilibrate the brain with a chamber temperature (CT) of −20°C and an object temperature (OT) of −16°C. Four 12 μm thick sections of the SON region were cut and placed on polyethylene naphthalate (PEN) Membrane Frame slides (Life Technologies, Grand Island, NY, Cat. # LCM0521) under RNAase-free conditions. Four brain sections were usually mounted on a single PEN membrane slide, and between 25 and 30 slides were made from a single brain specimen for up to 120 total sections. Slides were stored immediately in a slide box embedded in dry ice, and the slide box was placed in the −80°C freezer until the slides were used for LCM (slides were used within one month of being sectioned).

### Laser Capture Microdissection (LCM)

Slides were kept in their slide box on dry ice until the dehydration step for visualization on the LCM instrument. Before LCM, the sections were fixed and dehydrated with ethanol and xylene under RNase free conditions, as described previously with some modifications [30]. The slides were thawed for 30 seconds in 75% ethanol and further dehydrated by sequential immersion in 95% ethanol for 5 seconds, twice in 100% ethanol for 5 seconds, and followed by xylene for 30 seconds. The slides were left in a hood to allow the xylene to evaporate for 3 minutes. All solutions were made with DEPC water. Three slides can be placed in the Arcturus XT LCM instrument at one time, and therefore, only three slides were dehydrated for microdissection at one time. The rest of the slides were stored on dry ice until they were needed. The ethanol fixation technique was found to provide the best visualization of the SON under the LCM microscope (S1 Fig) and the highest RNA yield and quality (Table 1).

**Table 1:** Properties of Normosmotic and Salt-Loaded SON samples used in RNA-seq study^a^.

Condition	SON Sample	ng RNA/50ul	RIN
Normosmotic	HGN01	300	7.2
	HGN02	274	6.6
	HGN03	386	7.0
	Average	335	6.9
Salt-Loaded	HGSL01	658	7.6
	HGSL02	552	7.2
	HGSL03	748	7.2
	Average	652	7.3

^a^Each sample contains RNA from a single Rat’s SON pair. Ratio of average Salt-Loaded/Normosmotic SON’s ng RNA = 1.95

LCM was performed using the Arcturus XT (Life Technologies, Washington DC 20001) laser-microdissection system, immediately after fixation and dehydration of the slides. The SONs were microdissected using CapSure Macro LCM Caps (Life Technologies, Cat. # LCM0211). The PEN membrane frame slide was loaded on the instrument with the tissue side down and a clean glass slide underneath it acting as support [28]. The rat SON is completely visualized in the 20X objective view of the Arcturus XT LCM microscope which allowed for the microdissections of multiple SONs from many serial sections from the same brain specimen onto the same cap.

### RNA Isolation and Quality Assessment

The RNA was isolated using the Picopure RNA Isolation Kit (Life Technologies, Cat. #KIT0204). The Macro cap with the microdissected SONs was immediately seated into a 0.5 mL tube containing 50 μL of PicoPure Extraction buffer and incubated for 30 minutes at 42^°^C. When multiple caps for an individual rat were collected, the buffer from each cap containing the lysed cells was pooled in a 1.5 mL microcentrifuge tube before continuing with the isolation. After isolation, the RNA’s concentration was measured using a Nanodrop spectrophotometer (ND-1000, Thermo Scientific, Wilmington, DE). The RNA samples were analyzed using a 2100 Bioanalyzer (Agilent Technologies, Santa Clara, CA 95051) to obtain RNA integrity numbers (RIN numbers) as measures of their quality (Schroeder et al, 2006). Three RNA samples from Control SONs and three RNA samples from Salt-loaded SONs met our RIN quality criterion of >6.5, and these RNAs were taken for RNA-Seq analysis. See Table 1 for RNA yield and RIN numbers for these samples.

### RNA-Seq Methods

Amplified cDNA libraries were prepared from the isolated RNA samples shown in Table 1 and sequenced by the NIH Intramural Sequencing Center (NISC), Bethesda, MD under the direction of Baishali Maskeri and Alice Young. Briefly, mRNA libraries were constructed from 50–750 ng mRNA using the Illumina TruSeq RNA Sample Prep Kits, version 2. Equal quantities of the the resulting cDNAs for the individual control and SL samples were fragmented using a Covaris E210. Library amplification was performed using 12–15 cycles to minimize the risk of over-amplification. Unique barcode adapters were applied to each library. Equal volumes of individual libraries were pooled and run on a MiSeq. The libraries were then repooled based on the MiSeq demultiplexing results and sequenced on a HiSeq 2000 with ver 3 flow cells and sequencing reagents. At least 4x40 million 100-base read pairs were generated for each individual library. Data was processed using RTA 1.13.48 and CASAVA 1.8.2, providing for four sets of. fastq files per library. Reads contained within these files were subjected to sixteen analysis steps (See S2 Fig and legend). For the first analysis step, 3' adaptor sequence was removed from a read if present using the FASTQ/A Clipper tool (http://hannonlab.cshl.edu). For the second analysis step, reads were import into the CLCbio Genomics Workbench (www.clcbio.com) and quality inspected. For the third analysis step, 15nt from the 5' end of each read was globally trimmed in order to remove nucleotide bias and the last occurring nucleotide at the 3' end was removed due to low quality. Nucleotides with a call accuracy < 95% were also removed. While, read pairs with more than two ambiguities in at least one read were discarded along with pairs having at least one read < 15nt in length. For the fourth analysis step, remaining read pairs were aligned to the rat genome (rn5) for each library using the Workbench’s "RNA-Seq" tool under default parameters. For the fifth analysis step, the number of aligned read pairs to each known gene for each library were exported from the Workbench and imported into R (http://www.r-project.org/). In R, the number of aligned read pairs per known gene per library were converted to "Reads Per Kilo base of transcript Million mapped reads" ("RPKM") values. These RPPKM values were then pedestalled by a value of two (RPKM+2) to control for low-end variance. For the sixth analysis step, pedestalled RPKM values were transformed (Log2(RPKM+2) to promote normality then used to identify and remove non-informative genes (i.e., those not having at least one library with a transformed RPKM value >1). For the seventh analysis step, transformed RPKM values were quantile normalized (Quantile(Log2(RPKM+2))) to correct for library-to-library differences in distribution location, spread, and skew. For the eighth analysis step, normalized RPKM values were explored across libraries via Tukey box plots, covariance-based principal component analysis (PCA) scatter plot, and Pearson correlation-based heat maps to ensure absence of outliers. For the ninth analysis step, normalized RPKM values were organized by library class (i.e., salt-loaded control) and the coefficient of variation (CV) and mean normalized RPKM were calculated for each gene. Locally weighted scatterplot smoothing (LOWESS) was then applied by library class (CV ~ mean normalized RPKM) and the resulting fits plotted. For the tenth analysis step, the LOWESS fits were visually inspected to identify the mean normalized RPKM at which the linear relationship between mean normalized RPKM (i.e., signal) and CV (i.e., noise) is grossly and concordantly lost. This mean normalized RPKM value was then defined as the “confidence criterion”. For the eleventh analysis step, genes not having at least one library with a normalized RPKM greater than the "confidence criterion" were discarded as noise-biased. Normalized RPKM values for remaining genes were floored to equal the "confidence criterion" value if less. For the twelfth analysis step, the Welch-modified t-test was applied to the post-floored RPKM data on a gene-by-gene basis under Benjamin–Hochberg (BH) False-Discovery Rate (FDR) Multiple Comparison Correction (MCC) condition. This provided for one corrected p-value per gene. This resulting p-value describes the probability that the mean difference occurring between the library classes is due to chance. For the thirteenth analysis step, genes were filtered to keep only those having a corrected p-value < 0.10 and an absolute difference of means between library classes >/ = 1.5X. Genes kept were deemed to be those having differential expression between salt-loaded and control. For the fourteenth analysis step, floored RPKM values for these differential expressed genes were used in the generation of a covariance-based principal component analysis (PCA) scatter plot and Pearson correlation-based heat map to confirm good intra and inter library grouping by class. For the fifteenth and sixteenth analysis steps, the Ingenuity Pathway Analysis (IPA) tool (www.ingenuity.com) was used. Specifically, symbols for the genes deemed to have differential expression between salt-loaded and control were import into IPA and the corresponding enriched pathways and functions identified (uncorrected Fishers Exact Test p-value < 0.05).

**Fig 1:**
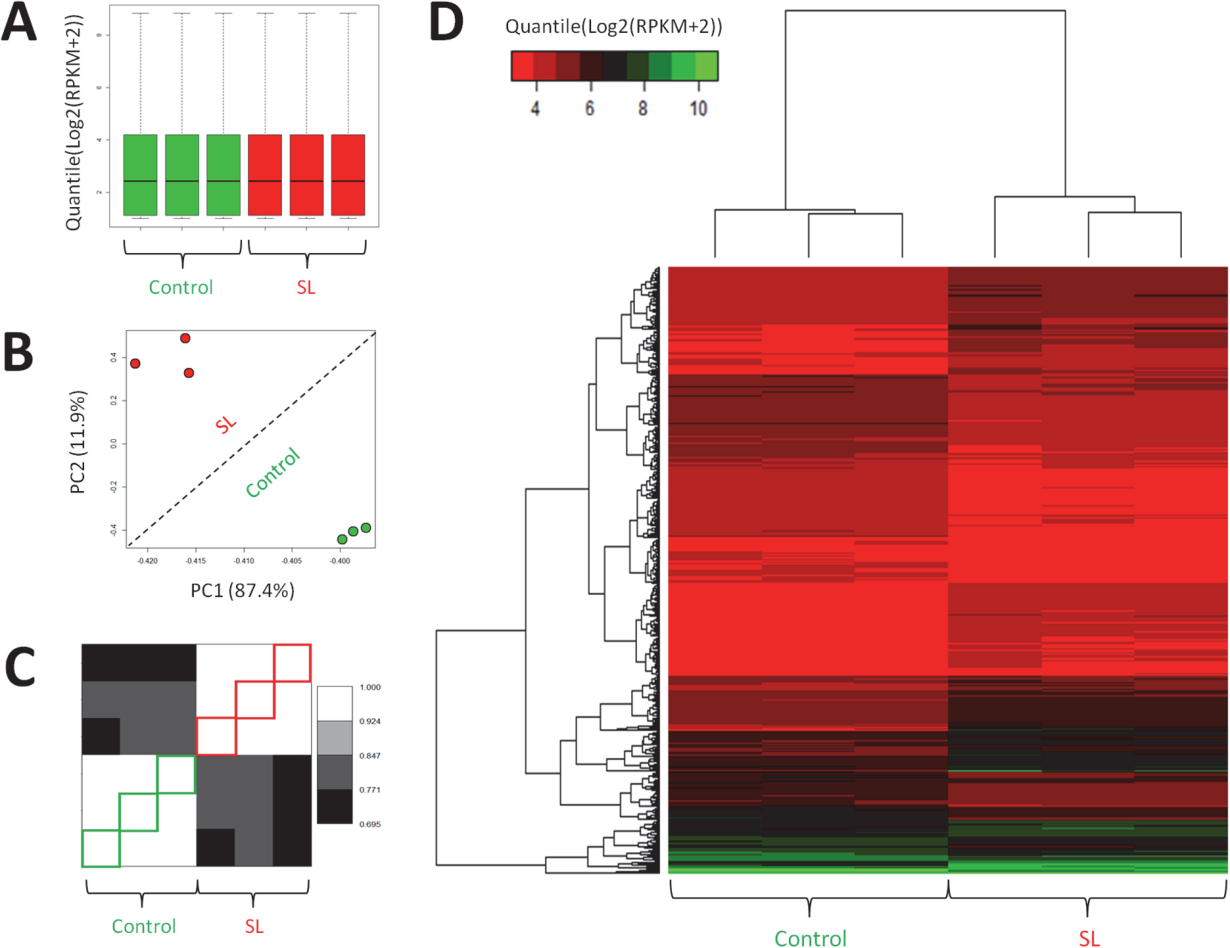
RNA-Seq Analysis Results. (A) Tukey box plot comparing the sample level distributions of gene expression data post normalization (Quantile(Log2(RPKM+2))) for 6 samples (3 controls, green-filled; 3 salt-loaded samples, red-filled). Distributions depicted consist of 26,313 gene expression measurements each. (B) covariance-based Principal Component Analysis (PCA) scatter-plot depicting relationships across 6 samples (3 controls, green-filled; 3 salt-loaded samples, red-filled) when normalized gene expression data (Quantile(Log2(RPKM+2))) is used for the 552 genes which are identified to have significant differential expression between salt-loaded (SL) condition and control. (C) correlation-based unclustered Heat-Map depicting relationships across 6 samples (3 controls, green-outlined; 3 salt-loaded samples, red-outlined) when normalized gene expression data (Quantile(Log2(RPKM+2))) is used for 552 genes identified to have significant differential expression between salt-loaded (SL) condition and control. (D) correlation-based clustered Heat-Map depicting relationships across 6 samples (3 controls, green-outlined; 3 salt-loaded samples, red-outlined) when normalized gene expression data (Quantile(Log2(RPKM+2))) is used for 552 genes identified to have significant differential expression between salt-loaded (SL) condition and control. Results reveal absence of outliers and excellent within and between sample class grouping.

Fig 1 describes the quality of the RNA-Seq data. There is good separation of the control and SL samples and Fig 1A illustrates the uniformity of the distribution of expression which indicates no sample outliers. Fig 1B illustrates the good within and between sample level groupings indicating neither experiment-level nor cohort-level outliers based on magnitude of expression. Fig 1C corroborates conclusions in 1B but is based on pattern of expression, and Fig 1D shows a heat map based on clustered differential expression.

Raw fastq files for all libraries can be found in the Short Read Archive (SRA; http://www.ncbi.nlm.nih.gov/Traces/sra/; SRP049482).

### Microarray Methods

CEL files were generated by the Affymetrix AGCC program for two separate microarray batches, one file per library, These files were subjected to twelve analysis steps (See S4 Fig and legend). For the first analysis step, all files were imported into the Affymetrix Expression Console (http://www.affymetrix.com) and Robust Multi-Array (RMA) expression summarization and normalization performed. This provided Log2 expression values for 31,099 gene fragments per library. For the second analysis step, expression data for all libraries was imported into R (http://cran.r-project.org/) and baseline subtraction performed to correct for microarray batch differences using the common library run between batches. For the third analysis step, batch-corrected expression values by library were explored via Tukey box plots, covariance-based principal component analysis (PCA) scatter plots, and Pearson correlation-based heat map to ensure absence of outliers. For the fourth analysis step, batch-corrected expression values were organized by library class (i.e., salt-loaded, control) and the coefficient of variation (CV) and mean expression calculated for each gene fragment. Locally weighted scatterplot smoothing (LOWESS) was then applied by library class (CV ~ mean expression) and the resulting fits plotted. For the fifth analysis step, the LOWESS fits were visually inspected to identify the expression value at which the linear relationship between mean expression (i.e., signal) and CV (i.e., noise) is grossly and concordantly lost. This expression value was then defined as the “confidence criterion”. For the sixth analysis step, gene fragments not having at least one library with an expression value greater than the "confidence criterion" were discarded as noise-biased. Expression values for remaining gene fragments were floored to equal the "confidence criterion" value if less. For the seventh analysis step, the Welch-modified t-test was applied to the post-floored expression values on a gene fragment-by-gene fragment basis under Benjamin–Hochberg (BH) False-Discovery Rate (FDR) Multiple Comparison Correction (MCC) condition. This provided for one corrected p-value per gene fragment. This resulting p-value describes the probability that the mean difference occurring between the library classes is due to chance. For the eighth analysis step, gene fragments were filtered to keep only those having a corrected p-value < 0.05 and an absolute difference of means between library classes >/ = 1.5X. Gene fragments kept were deemed to be those having differential expression between salt-loaded and control. For the ninth analysis step, floored expression values for these differential expressed genes were used in the generation of a covariance-based principal component analysis (PCA) scatter plot and Pearson correlation-based heat map to confirm good intra and inter library grouping by class. For the tenth, eleventh, and twelfth analysis steps, the Ingenuity Pathway Analysis (IPA) tool (www.ingenuity.com) was used. Specifically, the gene fragments deemed to have differential expression between salt-loaded and control were imported into IPA and the corresponding gene symbols identified along with the enriched pathways and enriched functions for those genes (uncorrected Fishers Exact Test p-value < 0.05). Comparison and intersection of results with those obtained by RNA-Seq was accomplished using the IPA-assigned gene symbol.

Raw. CEL files for all microarrays can be found in the Gene Expression Omnibus (GEO; http://www.ncbi.nlm.nih.gov/geo/; WD = GSE3110, SL = GSE65663).

### Immunohistochemistry

In separate experiments involving immunohistochemical assays, animals were anesthetized with isoflurane and immediately perfused transcardially with 100 ml of 0.1 M PBS at 10ml/min followed by 200ml of 4% paraformaldehyde (PFA) in 0.1 M PBS, PH 7.4 at 10ml/min. Brains were removed and, if they were soft and pink following the perfusion, they were post-fixed in 1% PFA overnight. Brains were then cryoprotected in increasing concentrations of sucrose in 0.9% saline. Sucrose concentrations were 5%, 10%, and 15%.

Coronal sections (16μm) were made on a cryostat (Reichert-Jung 2800; Frigocut, Heidelberg, Germany) and mounted onto coated slides (Fisher Scientific 12-550-19, Pittsburgh, PA). The sections were rinsed with 0.3% Triton-X in 0.1M PBS for 5 minutes, followed by two rinses of PBS of 5 minutes each. The sections were then incubated overnight at 4°C with a pan-specific anti-neurophysin mouse monoclonal antibody, PS 45, [31,32] at a dilution ratio of 1:100 in 0.1M PBS. After incubation in primary antibody the slides were placed on a shaker table at room temperature for 2 hours, rinsed 3 times with 0.1M PBS, and then incubated in secondary antibody, Alexa Flour 594-conjugated goat anti-mouse (Molecular Probes, Eugene, OR) at 1:500 dilution for two hours. After completing the immunohistochemistry, slides were counterstained with DAPI to detect all the nuclei in the section, rinsed three times in 0.1M PBS, and then coverslipped using Prolong Gold Antifade reagent (Molecular Probes) as a mounting medium.

In order to visualize the stained sections an epifluorescent microscope (Nikon Eclipse E400) was used. Representative images were then recorded using the accompanying camera (Q-Imaging RETIGA EXi Fast, Cooled Mono 12-bit). Optimizing the exposure time of the photograph to best display the representative images was done using Image-Pro Plus 5.1 (MediaCybernetics, Inc) imaging software. An illustration of an SON section showing PS 45 positive MCN cells, and MCN and non-MCN positive DAPI stained cells is shown in S3 Fig, and quantitative comparisons of these phenotypes is shown in S1 Table.

### Quantitative PCR validation of targets

Quantitative PCR methods were as described previously [33]. Brains from both the euhydrated and the 5-day salt loaded rats were carefully extracted and flash-frozen with powdered dry ice. The brain was mounted and 60μm sections stained with Toluidine blue (Sigma Aldrich; 0.1% in ethanol (70%, w/v) were made in order to identify the SON. Once localized, 1mm bilateral punches of the SON were taken from unstained tissue and stored in RNase-free tubes at -80°C.

When all samples were prepared, 1mL QIAzol lysis reagent (Qiagen) was added to each tube and left at room temperature for 5 minutes followed by centrifugation (10 minutes, 10,300rpm, 4 °C) in order to remove cellular debris following lysis. Chloroform was then added to the supernatant homogenate and the samples were centrifuged (15 minutes, 11,200 rpm, 4 °C) in order to extract the nucleic material. Total RNA was then precipitated using one volume of ethanol (70% w/v) and purified using the Qiagen RNeasy Mini Kit (Qiagen) according to manufacturers protocol. Using the QuantiTect Reverse Transcription Kit (Qiagen), cDNA was then prepared for qPCR assessment using primers according to S3 Table (constructed by MWG Eurofins unless otherwise stated) using the Applied Biosystems StepOnePlus Real-Time PCR System and fold-change assessed by establishing the ΔΔCt between the Rpl19 calibrator gene and the target gene.

## Results

### Characterization of the laser microdissected SON

The SON is composed of a single neuronal phenotype, the MCNs, which are large (20–40 um) neurons easily visualized in the Arturus XT LCM microscope (see S1 Fig). The MCNs are further divided into two subtypes, the Oxt- and Avp-MCNs with the Avp neurons predominating in an approximately 2:1 ratio in the SON [2,34]. MCNs are the primary sources of the RNA isolated from the laser microdissected SON. However, there is also a non-neuronal small cell (e.g, glial, vascular, etc.) population in the SON that can be visualized by DAPI-staining of all nuclei in the SON (S3 Fig). By quantitatively comparing the small dark-stained nuclei in the small cell population to the larger, lighter DAPI stained nuclei in the immunohistochemically identified MCNs in the SON we estimate that there are twice as many non-neuronal cells than MCNs in the SON (S1 Table).

Table 2 shows the quantitative determination of all the phenotype-specific RNAs found by the RNA-Seq analysis of the microdissected SONs presented in both quantile normalized Log2(RPKM+2) units and in relative gene expression linear units (in parentheses). The most abundant transcripts expressed in the SON are the the Oxt-and Avp peptide RNAs, whose expression is orders of magnitude greater than the relatively abundant pan-neuronal specific beta tubulin 3 (TUBB3) gene. The Avp RNA expression is about two fold greater than the Oxt-RNA expression in the SON consistent with the greater number of Avp MCNs as compared to Oxt MCNs present in the SON. Other MCN-specific marker genes such as prodynorphin (Pdyn), tyrosine hydroxylase (TH), and the pan-neuron-specific genes such as TUBB3 and neurofilament M (NEFM) are also highly expressed in the SON.

**Table 2:** Relative Expression of Cell-Type Specific Markers in SON Samples^a^.

Phenotype	Gene Symbol	Normosmotic SON	Salt-Loaded SON	Fold Change (SL/N)
MCN	OXT	13.64 (12,766)	13.37 (10,587)	-
	AVP	14.79 (28,329)	14.79 (28,329)	-
	PDYN	9.68 (820)	10.57 (1,520)	1.9^b^
	TH	5.36 (42)	8.55 (388)	9.1^b^
	VGF	6.70 (104)	8.70 (416)	3.9^b^
Neuron	TUBB3	9.02 (519)	9.40 (676)	1.3^b^
	GAP43	4.68 (26)	5.24 (38)	1.5^b^
	NEFM	3.90 (15)	4.37 (21)	1.4^b^
Astrocyte	GFAP	8.29 (313)	7.18 (145)	-2.3^b^
	S100b	8.34 (313)	7.57 (190)	-1.7^b^
	Aldhi	4.10 (17)	3.48 (11)	-1.4^b^
Microglia	AIf1 (Iba1)	3.03 (8)	3.03 (8)	1
Oligodendroglia	MBP	5.79 (55)	5.45 (45)	-1.3
	MOG	4.32 (20)	4.33 (20)	1
Blood Vessel	PeCam1	3.90 (15)	4.08 (17)	1.1
	Procr	3.00 (8)	6.70 (104)	12.8
Non-specific	Gapdh	10.97 (2,006)	10.91 (1,924)	-1.04
	Actin, beta	10.16 (1,144)	9.90 (953)	-1.2

^a^Data shown are taken from S4 Table, are averages of three samples each and are expressed in quantile (log2 (RPKM+2)) units. The numbers in parentheses represent the relative gene expression in linear units (i.e, the antilog of the log2 values). Note that the relative values range from 8 (barely above the noise level) for AIf1 to 28, 239 for AVP, covering a range of expression of over four orders of magnitude. AVP expression is 55-fold greater than the pan-neuronal cytoskeletal protein marker, TUBB3, and the TUBB3 is 1.7 fold greater than the astrocytic cytoskeletal protein marker, GFAP, expression in the SON.

Abbreviations: AVP, arginine vasopressin; OXT, oxytocin; PDYN, prodynorphin; TH, tyrosine hydroxylase; VGF, nerve groth factor inducible (in secretogranin/chromogranin family); TUBB3, beta tubulin 3; GAP43, Growth Associated Protein 43; NEFM, neurofilament protein M; GFAP, glial fibrillary acidic protein; S100b, S100 calcium binding protein B; Aldhi, aldehyde dehydrogenase 2 family (mitochondrial); AIf1 (Iba1), ionized calcium-binding adapter molecule 1; MBP myelin basic protein; MOG, myelin oligodendrocyte glycoprotein; PeCam1, Platelet endothelial cell adhesion molecule; Procr, protein C receptor.

^b^statistically significant (p< 0.05) fold changes (in SL versus Control conditions).

As expected, various non-neuronal cell (e.g, astrocyte, microglial, vascular, etc.) RNAs are also found in the SON sample but at much lower levels. The presence of oligodendroglial-specific RNAs such as myelin basic protein (MBP) RNA could represent a slight tissue contamination from the optic chiasm adjacent to the SON, but is more likely due to the detection of sequences of a related but distinct neuronal protein known as golli-MBP [35,36], previously shown to be significantly expressed in the SON [29].

### RNA-Seq Analysis of the SON in control (normosmotic) and SL conditions

A systematic description of our RNA-Seq analysis is presented in S2 Fig (and in Methods). A critical component in this analysis is our approach to set the noise threshold level of RNA-Seq gene expression (see S5A Fig) at or below which no data was accepted as reliable. This meant that most of the data obtained for the 26,313 genes that were detected by the RNA-Seq method before noise level correction were removed from consideration, and in this way probably increased the reliability of the positive data but also increased the likelihood of false negatives. Following noise correction the genes found to be present in the Control and SL SON transcriptomes are shown in S4 Table and S5 Table, respectively. S6 Table presents the total number of 9,709 genes that were expressed in both the control the SL SONs, and S7 Table provides the statistical testing data for these 9,709 genes. Thus, the data in S6 and S7 Tables represents the most reliable identification of the genes that are present in the SON, provides their ingenuity descriptions, and shows their fold changes in expression in response to SL. While these genes are all definitely present in the SON, few significantly change in expression as a result of SL. An example of this is shown in S8 Table which shows that of the 772 of the genes found expressed in the SON and involved in either transcriptional or translational processes (in S7 Table), only about 42 are significantly changed in gene expression by SL The transcriptional and translational regulator genes that do significantly change in gene expression by SL are shown in Tables 3 and 4. Table 3 lists those genes that increase and Table 4 lists those that decrease in expression after SL. Another functional class of genes related to transcriptional regulation are the ligand-dependent nuclear receptor family of genes. A list of the nuclear (hormone) receptor mRNAs that are found in the SON and their changes in gene expression is presented in Table 5. Here too, there were only modest, if any, changes in gene expression of the ligand-dependent nuclear receptors produced by SL. In contrast, Tables 6 and 7 list a wide variety of other genes with diverse functions that are significantly and strongly increased and decreased, respectively, in expression by SL (with greater than two-fold changes).

**Table 3:** Transcriptional and Translational Regulator mRNAs increased in expression by SL.

Gene Symbol	Mean Control^a^	Mean SL^a^	Fold Change	Corrected P	Ingenuity Description
Creb3l1	4.16	6.87	6.54	0.034	cAMP responsive element binding protein 3-like 1
Eif4ebp1	3.47	5.44	3.92	0.047	eukaryotic translation initiation factor 4E binding protein 1
Eaf1	3.20	4.98	3.42	0.039	ELL associated factor 1
Atf4	7.68	9.34	3.18	0.031	activating transcription factor 4
Atf5	5.54	7.06	2.87	0.028	activating transcription factor 5
Nab1	3.65	4.96	2.48	0.028	NGFI-A binding protein 1 (EGR1 binding protein 1)
Etv5	3.35	4.60	2.38	0.062	ets variant 5
Hdac9	3.78	4.97	2.28	0.056	histone deacetylase 9
Htatip2	3.33	4.34	2.00	0.036	HIV-1 Tat interactive protein 2, 30kDa
Dap	5.14	6.08	1.93	0.034	death-associated protein
KLF6	3.29	4.10	1.75	0.078	Kruppel-like factor 6
Tle1	3.43	4.23	1.74	0.059	transducin-like enhancer of split 1 (E(sp1) homolog, Drosophila)
Ankrd55	3.80	4.58	1.72	0.074	ankyrin repeat domain 55
DDIT3	5.34	6.09	1.69	0.036	DNA-damage-inducible transcript 3
Eif1a	5.62	6.34	1.64	0.028	eukaryotic translation initiation factor 1A, Y-linked
Litaf	3.01	3.73	1.64	0.079	lipopolysaccharide-induced TNF factor
Cebpg	4.46	5.10	1.56	0.031	CCAAT/enhancer binding protein (C/EBP), gamma
Maged1	8.46	9.10	1.56	0.073	melanoma antigen family D, 1
Pir	4.32	4.95	1.55	0.053	pirin (iron-binding nuclear protein)
Smyd1	4.46	5.08	1.54	0.036	SET and MYND domain containing 1
Elk3	3.13	3.73	1.51	0.085	ELK3, ETS-domain protein (SRF accessory protein 2)

^a^Mean Control and Mean SL data are averages of three samples each and are expressed in quantile (log2 (RPKM+2)) units.

**Table 4:** Transcriptional and Translational Regulator mRNAs decreased in expression by SL.

Gene Symbol	Mean Control^a^	Mean SL^a^	Fold Change	Corrected P	Ingenuity Description
Dbp	4.51	3.07	-2.72	0.025	D site of albumin promoter (albumin D-box) binding protein
Tef	4.41	3.43	-1.97	0.039	thyrotrophic embryonic factor
Cirbp	5.59	4.69	-1.87	0.038	cold inducible RNA binding protein
Pou3f2	6.28	5.47	-1.76	0.065	POU class 3 homeobox 2
Mzf1	3.81	3.01	-1.75	0.068	myeloid zinc finger 1
ANKRD10	6.33	5.54	-1.73	0.059	ankyrin repeat domain 10
Zfp483	4.18	3.43	-1.68	0.054	zinc finger protein 483
Id3	5.74	5.03	-1.64	0.084	inhibitor of DNA binding 3, dominant negative helix-loop-helix protein
Hdac11	6.60	5.89	-1.63	0.038	histone deacetylase 11
Rbck1	5.15	4.44	-1.63	0.028	RanBP-type and C3HC4-type zinc finger containing 1
Anks3	5.12	4.42	-1.63	0.047	ankyrin repeat and sterile alpha motif domain containing 3
Ldb2	5.13	4.43	-1.62	0.028	LIM domain binding 2
Pqbp1	7.42	6.75	-1.60	0.058	polyglutamine binding protein 1
Zfp187	4.69	4.03	-1.57	0.040	zinc finger and SCAN domain containing 26
Heyl	4.36	3.75	-1.53	0.034	hes-related family bHLH transcription factor with YRPW motif-like
Abtb1	4.11	3.50	-1.52	0.061	ankyrin repeat and BTB (POZ) domain containing 1
Calcoco1	4.89	4.28	-1.52	0.053	calcium binding and coiled-coil domain 1
Hdac10	4.78	4.18	-1.51	0.082	histone deacetylase 10
Deaf1	4.33	3.74	-1.51	0.040	DEAF1 transcription factor
Hes6	3.79	3.20	-1.51	0.033	hes family bHLH transcription factor 6
Maz	4.76	4.17	-1.50	0.031	MYC-associated zinc finger protein (purine-binding transcription factor)

^a^Mean Control and Mean SL data are averages of three samples each and are expressed in quantile (log2 (RPKM+2)) units.

**Table 5:** Ligand-dependent nuclear receptor mRNAs expressed in SON.

Gene Symbol	Mean Control^a^	Mean SL^a^	Fold Change	Corrected P	Ingenuity Description
Nr4a1	3.48	3.74	1.19	0.680	nuclear receptor subfamily 4, group A, member 1
Esrra	3.69	3.84	1.11	0.178	estrogen-related receptor alpha
Ppard	2.97	3.07	1.07	0.231	peroxisome proliferator-activated receptor delta
Nr1i3	4.42	4.48	1.05	0.564	nuclear receptor subfamily 1, group I, member 3
Nr1h2	4.04	4.03	-1.00	0.932	nuclear receptor subfamily 1, group H, member 2
Nr2f2	4.00	3.94	-1.04	0.752	nuclear receptor subfamily 2, group F, member 2
Nr2c2	3.12	3.06	-1.04	0.476	nuclear receptor subfamily 2, group C, member 2
Nr3c1	3.04	2.96	-1.06	0.068	nuclear receptor subfamily 3, group C, member 1 (glucocorticoid receptor)
Nr2f6	3.63	3.52	-1.08	0.367	nuclear receptor subfamily 2, group F, member 6
Nr2f1	4.89	4.74	-1.11	0.211	nuclear receptor subfamily 2, group F, member 1
Dnttip1	4.42	4.23	-1.14	0.246	deoxynucleotidyltransferase, terminal, interacting protein 1
Rxra	3.73	3.52	-1.16	0.359	retinoid X receptor, alpha
Rxrg	3.37	3.00	-1.29	0.248	retinoid X receptor, gamma
Nr1d1	3.76	3.37	-1.31	0.113	nuclear receptor subfamily 1, group D, member 1
Thra	7.23	6.84	-1.31	0.058	thyroid hormone receptor, alpha
Rxrb	4.48	3.95	-1.45	0.076	retinoid X receptor, beta
NR1D2	3.75	3.00	-1.67	0.067	nuclear receptor subfamily 1, group D, member 2

^a^Mean Control and Mean SL data are averages of three samples each and are expressed in quantile (log2 (RPKM+2)) units.

**Table 6:** Other mRNAs in SON that are increased in expression by SL.

Gene Symbol	Mean Control^a^	Mean SL^a^	Fold Change	Corrected P	Ingenuity Description	Ingenuity_Type
Vgf	6.71	8.69	3.93	0.031	VGF nerve growth factor inducible	growth factor
Slc7a3	4.61	6.39	3.43	0.040	solute carrier family 7 (cationic amino acid transporter, y+ system), member 3	transporter
Gpr88	3.03	4.71	3.22	0.069	G protein-coupled receptor 88	G-protein coupled receptor
Ldlr	3.34	4.86	2.88	0.052	low density lipoprotein receptor	transporter
Rxfp3	4.29	5.65	2.58	0.060	relaxin/insulin-like family peptide receptor 3	G-protein coupled receptor
Opn3	5.69	7.02	2.51	0.081	opsin 3	G-protein coupled receptor
Cckbr	3.01	4.34	2.51	0.053	cholecystokinin B receptor	G-protein coupled receptor
Fabp7	6.55	7.86	2.48	0.049	fatty acid binding protein 7, brain	transporter
Kcnk1	3.53	4.83	2.46	0.042	potassium channel, subfamily K, member 1	ion channel
Slc24a3	4.20	5.49	2.44	0.047	solute carrier family 24 (sodium/potassium/calcium exchanger), member 3	transporter
Trpc4	3.00	4.19	2.28	0.070	transient receptor potential cation channel, subfamily C, member 4	ion channel
Gpr158	3.99	5.15	2.23	0.045	G protein-coupled receptor 158	G-protein coupled receptor
Slc5a10	3.01	4.15	2.20	0.065	solute carrier family 5 (sodium/sugar cotransporter), member 10	transporter
Fabp3	5.59	6.72	2.18	0.052	fatty acid binding protein 3, muscle and heart (mammary-derived growth inhibitor)	transporter
Slc41a2	3.76	4.85	2.13	0.034	solute carrier family 41 (magnesium transporter), member 2	transporter
Mchr1	3.04	4.10	2.09	0.082	melanin-concentrating hormone receptor 1	G-protein coupled receptor
Fxyd5	5.54	6.60	2.09	0.083	FXYD domain containing ion transport regulator 5	ion channel
Trpv2	4.29	5.31	2.03	0.037	transient receptor potential cation channel, subfamily V, member 2	ion channel

^a^Mean Control and Mean SL data are averages of three samples each and are expressed in quantile (log2 (RPKM+2)) units.

**Table 7:** Other mRNAs in SON that are decreased in expression by SL.

Gene Symbol	Mean Control^a^	Mean SL^a^	Fold Change	Corrected P	Ingenuity Description	Ingenuity Type
Fxyd6	6.79	5.20	-3.02	0.028	FXYD domain containing ion transport regulator 6	ion channel
NNAT	10.20	8.84	-2.56	0.083	Neuronatin (PEG5)	transporter
Agt	8.16	6.87	-2.46	0.064	angiotensinogen (serpin peptidase inhibitor, clade A, member 8)	growth factor
Hba1	7.57	6.29	-2.43	0.095	hemoglobin, alpha 1	transporter
Ntsr2	5.32	4.07	-2.38	0.038	neurotensin receptor 2	G-protein coupled receptor
Hpcal4	5.30	4.06	-2.37	0.045	hippocalcin like 4	transporter
Slc17a4	4.36	3.15	-2.31	0.084	solute carrier family 17, member 4	transporter
ENSRNOG00000031230	4.48	3.30	-2.28	0.076	hemoglobin, beta	transporter
Fxyd1	4.44	3.31	-2.19	0.053	FXYD domain containing ion transport regulator 1	ion channel
Rbp1	4.30	3.26	-2.06	0.065	retinol binding protein 1, cellular	transporter
Gprc5b	6.18	5.15	-2.03	0.036	G protein-coupled receptor, family C, group 5, member B	G-protein coupled receptor

^a^Mean Control and Mean SL data are averages of three samples each and are expressed in quantile (log2 (RPKM+2)) units.

**Fig 2:**
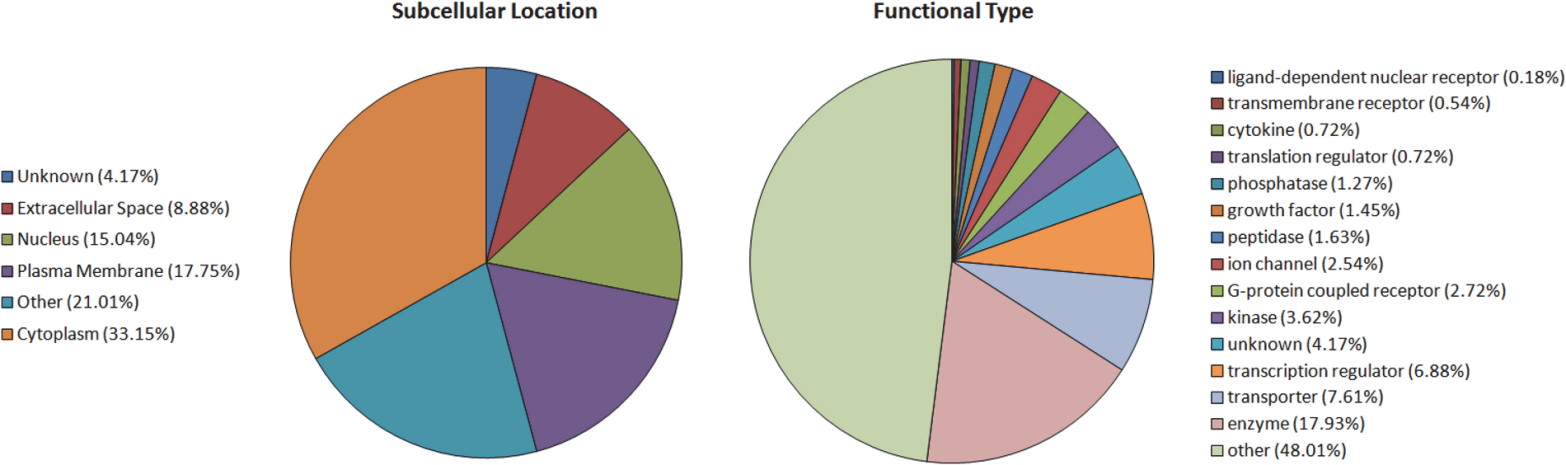
Pie Chart showing Locations and Types of 552 selected, expressed genes (See columns N and O in S9 Table).

Further statistical analysis of the RNA-Seq data shown in S7 Table is presented in S9 Table where 552 genes are selected as having the most reliable changes in expression as a result of SL treatment. Fig 2 shows the functional types and subcellular locations of these 552 genes in the SON.

### Microarray Analysis of the SON in control (normosmotic) and SL conditions

**Fig 3:**
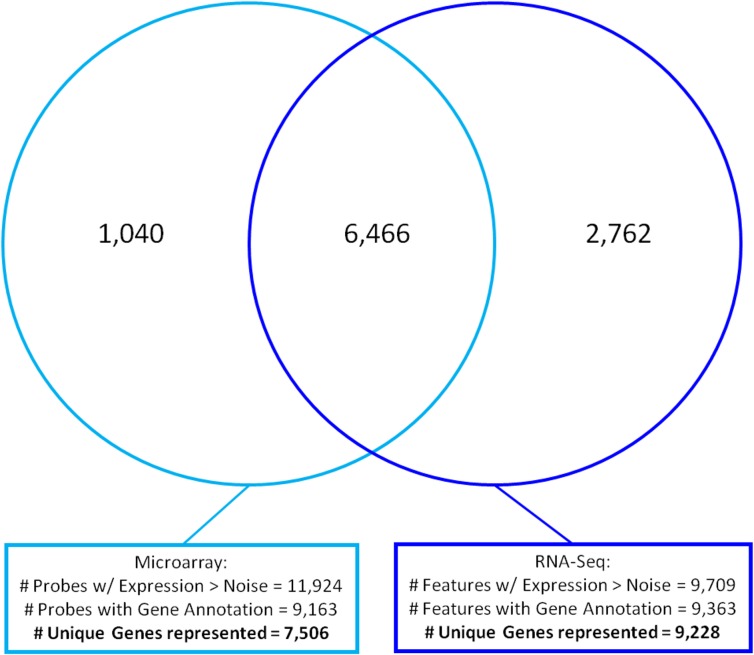
Venn diagram of intersections of RNA-Seq and Microarray data of post-noise filtered genes representing 7,506 microarray and 9,228 RNA-Seq genes. Complete data are shown in S4 Table for RNA-Seq genes and S10 Table for microarray genes. Note that 6,466 unique genes are found in both platforms to intersect.

The strategy used to analyze the microarray data is shown in S4 Fig (and Methods), and the analysis of the noise threshold for the microarray data is illustrated in S5B Fig. Using this approach, 10,382 gene probes were identified as being expressed in control SONs S10 Table), 10,476 gene probes in SL SONs (S11 Table), and 11,293 gene probes in both control and SL SONs (S12 Table). After conversion of the identified gene probes derived from the microarray analysis and the RNA-Seq data to gene symbols, the intersections of these data, based on their unique symbols, were determined and illustrated in the Venn diagram shown in Fig 3. There were 6,466 genes present in the SON that were represented in both the microarray and RNA-Seq data, however 1,040 of the expressed genes were found only in the microarray data, and 2,762 of the expressed genes were selectively found in the RNA-Seq data and not in the microarray data.

### Comparison of RNA-Seq and Microarray Analyses of specific gene expression changes in the SON as a result of SL

**Fig 4:**
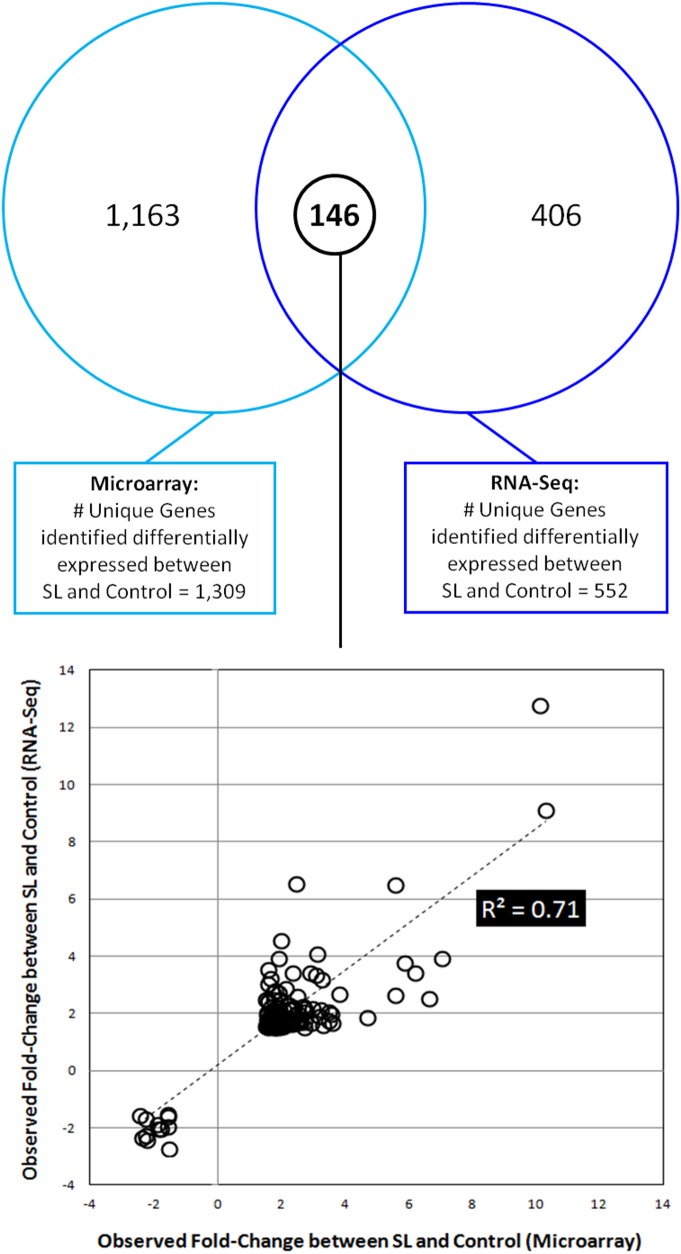
Venn diagram of intersections of RNA-Seq and microarray data after correcting for noise threshold and statistical analyses (see S2 Fig, Steps 11 and 12, and S4 Fig steps 6 and 7 for microarray). There are 552 genes selected from the RNA-Seq data (see S9 Table) and 1030 unique genes selected from the microarray analysis (S13 Table). Note that 146 unique genes are found from both platforms to be unequivocally expressed and altered by salt-loading (See S14 and S15 Tables). Lower panel: Shows X-Y scatter plot of the fold changes in the 146 genes observed between Con & SL samples that were found in both the Microarray (X-axis) and RNA-Seq (Y-axis) data.

Fig 4 depicts a Venn diagram display of the intersection of RNA-Seq and microarray data after correcting for noise thresholds and statistical analyses of each data set. The RNA-Seq data shown in Fig 4 is derived from the 552 identified genes presented in S9 Table, and the microarray data used is from the 1,309 identified gene probes in S13 Table. The 146 unique genes illustrated in Fig 4 whose expression in the SON is altered by SL were found to intersect between the two different platforms. These correspond to the 170 gene probes in the microarray study (S14 Table) and are listed in S15 Table as the 146 intersecting gene candidates. Fig 4 also shows that in addition to the 146 genes present in the SON that are represented in both data sets, there are 406 expressed genes that are altered in expression by SL that are selectively found in the RNA-Seq data but not in the microarray data (see S16 Table). In addition, there are 1139 expressed gene probes (S17 Table) which convert to 884 unique genes (Fig 4), that are altered in expression in the SON by SL and are found only in the microarray data.

### Ingenuity Pathway Analysis (IPA) of networks, enriched functions, enriched pathways for the 552 RNA-Seq genes selected as differentially expressed between SL and Control SONs

**Fig 5:**
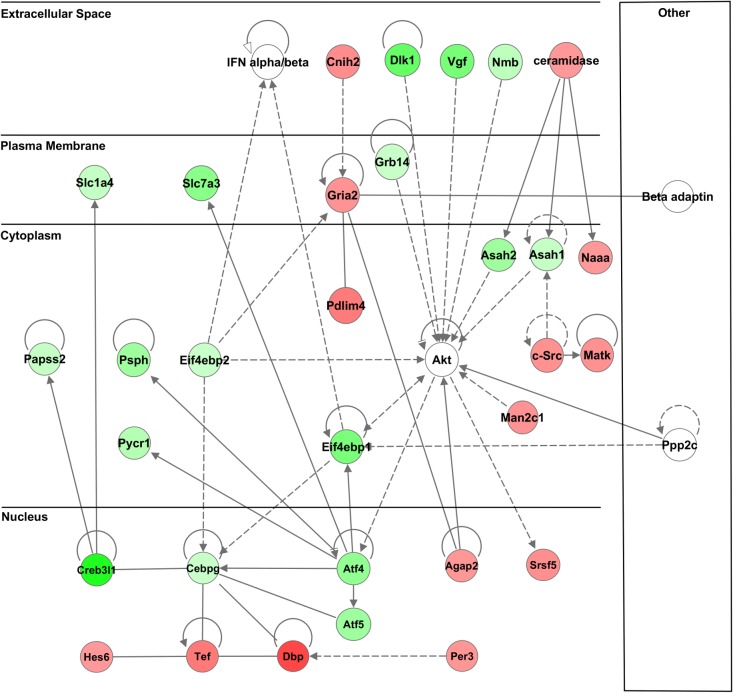
Network Analysis depicting gene products and known relationships between them for the second-ranked scoring network by Ingenuity (http://www.ingenuity.com/) when provided list of differentially expressed genes observed between salt-loaded and control by RNA-Seq (based on data in S9 Table). Gene products are represented using circle-shaped symbols with connected edges drawn between them to describe interactions (solid edge = direct interaction, dashed edge = indirect interaction). Color-filled shapes indicate the direction of differential expression observed between salt-loaded and control (green = up, red = down). Circle-shaped symbols not color-filled represent gene products not observed differentially expressed between salt-loaded and control.

Applying Ingenuity Pathway Analysis (IPA) (www.ingenuity.com) to the data shown in S9 Table suggests that certain functional relationships and interactions between the genes in the SON have been altered as a result of SL treatment. S18 Table shows the top 25 Networks proposed by IPA for the 552 RNA-Seq genes that were selected as differentially expressed between SL and Control. SONs. The top two predicted Networks are illustrated in Fig 5 (with Atf4 and Akt as hub genes) and in S6 Fig (with NFkB as a hub gene). Other IPA core analyses suggest predicted enriched functions (S19 Table) and enriched pathways (S20 Table) for the 552 RNA-Seq genes.

### qRT-PCR validations of selected genes in the SON

Table 8 summarizes the results of qRT-PCR data illustrated in S7 Fig for several genes that showed very large changes in expression between control and SL SONs in RNA-Seq (e.g, vgf and procr) and several genes that were identified in the network analysis (see Fig 5). Relevant RNA-Seq data are also shown for comparison in Table 8. Note that all but two genes (Insig1 and Eaf1) were confirmed by qPCR as being significantly regulated by salt loading treatment.

**Table 8:** Selected qPCR determinations of fold changes in gene expression due to SL in the SON.

Gene Symbol	qPCR		RNA-Seq		Ingenuity Description
	Fold Change^a^	P	Fold Change^b^	Corrected P	
vgf	9.5	0.006	3.9	0.03	VGF nerve growth factor inducible
Procr	26.3	0.02	12.8	0.07	protein C receptor, endothelial
Creb3l1	7	0.004	6.5	0.03	cAMP responsive element binding protein 3-like 1
Giot1 (Zfp932)	8.5	0.02	2.4	0.09	zinc finger protein 932
Atf4	2.2	0.006	3.2	0.03	activating transcription factor 4
Arhgdib	7.6	0.001	2.7	0.04	Rho GDP dissociation inhibitor (GDI) beta
Opsin3 (Opn3)	7.5	0.005	2.5	0.08	opsin 3
Atf5	2.7	0.004	2.9	0.03	activating transcription factor 5
cebpg	1.6	0.02	1.6	0.03	CCAAT/enhancer binding protein (C/EBP), gamma
Eaf1	1.3	0.08	3.4	0.04	ELL associated factor 1
Psph	2	0.0001	2.8	0.03	phosphoserine phosphatase
Nab1	1.9	0.009	2.5	0.03	NGFI-A binding protein 1 (EGR1 binding protein 1)
Insig1	1.2	0.21	3	0.03	insulin induced gene 1
Oacyl	1.7	0.08	6.5	0.09	O-acyltransferase like
Trpv2	3.1	0.01	2	0.04	transient receptor potential cation channel, subfamily V, member 2
hnVP1	2.9	0.02	—	—	Arginine vasopressin (hnRNA)
Oxt	2.5	8.90E-05	—	—	oxytocin

^a^Fold change was established using delta-delta-Ct method and the control normalized to its own mean (e.g. 1). Stats were done by a simple student t-test in excel.

^b^Fold change and corrected p-value from S7 Table.

### Alternatively Spliced Genes expressed in the SON under Control and SL conditions

The CLCbio Workbench (www.clcbio.com) was employed to search for evidence of alternative splicing in the RNA-Seq data that occurred during SL. RNA-Seq expression was generated for a total of 27,180 transcripts. After normalization, noise modeling, and filtering, there were data for 8,537 transcripts that underwent statistical testing. Expression levels differed significantly between the SL and control groups for 529 of the transcripts subjected to the IPA analysis. Of these 529 transcripts, 525 are represented by unique genes and 4 mapped to the same gene (2 were mapped to the Argn gene and 2 were mapped to the Nnat gene). This indicates a very modest extent of alternative splicing in the 529 evaluated SON genes. These data are shown in S21 Table.

Graphic representations of these data are shown in S8 Fig for Argn which has 38 exons and in S9 Fig for Nnat which has 3 exons. The only difference between the two Argn transcripts is that the final exon in this gene is missing from one of the sliced variants (see star in S8 Fig). S9 Fig shows that the middle exon (see star) is missing from one of the transcripts of Nnat. Both Nnat transcript forms are expressed in SL and control SONs: expression is higher under SL conditions, and the 2 exon form is more highly expressed than the 3 exon form.

## Discussion

RNA sequencing (“RNA-Seq”) is a powerful tool for obtaining quantitative information about the population of RNA species that are present in specific regions of microdissected brain tissue [37]. We utilized this approach to study the SON in rat hypothalamus in order to characterize the SON’s transcriptome under normal (isoosmotic) and chronic hyperosmotic conditions produced by salt loading (SL). The MCNs in the hypothalamus are among the few neuronal phenotypes in the CNS that respond to systemic osmotic perturbations by robust physiological changes [9,38] and changes in gene expression [7].We chose to study the SON since this brain region contains only two neuronal phenotypes, the Oxt and Avp MCNs [2], and many studies have previously been done that show large increases in expression of the principal neuropeptide genes, Oxt and Avp genes, in the MCNs in the SON during SL [7,39,40,41,42]. Moreover, chronic SL produces large increases in the volumes of the MCNs which are recognized to be due to global increases in transcription and protein synthesis in the MCNs under hyperosmotic stimulation [10,11,12,15]. Evidence for a global increase in gene expression in the SON during chronic hyperosmotic stimulation has also been obtained by microarray studies [16,17,18,19]. It should be reiterated here that while the MCNs are the only neuronal phenotypes in the SON, this nucleus is in fact heterogeneous, and also contains glial and vascular cellular phenotypes. Consequently, all of these cell types will contribute to the RNA species being expressed and measured in the SON under both normal (control) and SL conditions. When cell-type specific markers are known it is possible to unequivocally identify in which cells these specific RNAs are changing in response to SL (see Table 2). However, for most of the RNAs being sampled in the SON transcriptome identification of the specific cellular phenotypes in the SON responsible for the expression changes will require the use of additional complementary methods such as single cell qPCR [43,44] or quantitative in situ hybridization [45,46].

In this paper, we present for the first time RNA-Seq evidence for an increase in global gene expression in the SON in response to chronic SL. There are many advantages for using RNA-Seq to characterize the transcriptome in a given tissue [47,48]. RNA-Seq profiles the transcriptome by deep profiling of isolated RNAs, and hence, provides an unbiased approach to study the gene expression of known and novel RNA transcripts, and can also reveal alternative isoforms. Transcript abundance is directly proportional to the number of reads that map to a specific transcript, and RNA-Seq has a large dynamic, linear range [47,49]. In this paper, we also compare data obtained from RNA-Seq and microarray platforms, and as it has been pointed out by others [48] the overlap is greatest for the highest expressed genes. Genes expressed at the lowest levels of detectability on microarrays are often not detected by RNA-Seq largely because of insufficient sequencing depth often used for the latter method [48]. Several studies have been done which compare microarray and RNA-Seq and while most argue for the preference of RNA-Seq for the elucidation of transcriptomes [48,49,50], there are also limitations to the use of RNA-Seq for these purposes [49].

### The SON Transcriptome and Effects of SL

RNAs representative of all of the cellular phenotypes expected to be present in the SON are present in the RNA-Seq data obtained from the SON (Table 2), and the MCN- and astrocyte-specific RNAs are predominant in the SON samples. Both the MCN- and astrocyte-specific RNA expressions are significantly altered by SL treatment, but in opposite directions. All the MCN-specific and pan-neuronal RNA markers were specifically and substantially increased by SL, which is comparable to the two-fold increase in total RNA levels in the SON produced by SL (S2 Table). In contrast to the MCN-specific transcripts, all of the astrocyte RNA markers (e.g, GFAP and S100b) were significantly decreased in expression under SL conditions (Table 2). This increase in expression of neuronal markers together with a decrease in expression of astroglial markers such as GFAP are similar to that previously reported in the SON of dehydrated rats [51]. None of the other non-neuronal marker RNAs shown in Table 2 showed statistically significant changes in expression levels with SL. The extremely high levels of the Oxt- and Avp- RNAs in the RNA-Seq data compromised our ability to accurately quantify the changes in expression of these genes during SL. Such high levels of expression produce difficulties in RNA-Seq analysis which are described as transcriptional amplification [52]. However, it is well established that Oxt- and Avp gene expression approximately doubles in the SON during chronic SL [7] and this was confirmed by the qPCR data obtained in this study (see Table 8).

Our RNA-Seq analysis reliably detected 9709 genes in the SON samples. These data for the SON transcriptome are presented in S7 Table together with their fold changes in SL, their Ingenuity symbols and descriptions of the listed genes. Since the SON contains non-neuronal cell phenotypes in addition to the MCNs, it is important to note that the GFAP marker molecule for the most abundant non-neuronal phenotype, the astrocytes, contains one hundreth of the number of transcripts than that found for the Avp mRNAs in the SON (Table 2). As noted above, the MCN-specific markers and pan-neuronal mRNAs increased with SL, whereas the opposite, a decrease in mRNA was found for the astrocytic markers (e.g, GFAP, in Table 2). The abundance of the mRNAs from the other non-neuronal phenotypes (e.g. microglia, oligodendroglia, vascular) were very low and close to the noise level, and showed no significant changes as a result of SL. Therefore, it is likely that most of the increases in gene expression that are found with SL are due to changes within the MCNs. The 552 genes that show statistically significant changes in SL are listed in S9 Table. These 552 genes represent a variety of functions and subcellular locations (Fig 2). Some of these changes are in transcriptional and translational regulator genes (Table 3) and various other genes that were changed more than two-fold by SL treatment (Table 5). The fold changes found in a selected number of genes in this group were confirmed by qPCR (Table 8).

The microarray analysis identified 7,506 genes that were expressed in both control and SL SONs (S12 Table). Comparisons of the unique genes predicted by the microarray data and the genes predicted from the RNA-Seq data to be present in the SON is shown in the Venn diagram in Fig 3. There are 6,466 genes that intersect between these different platforms, and 1,040 genes were found only in the microarray data, and 2,762 genes in the RNA-Seq data were not present in the microarray data (Fig 3). Similar differences in comparisons between RNA-Seq and microarray data analyses of the same experiments have been reported in other studies [48,53]. These differences also may be due, in part, to the difference in sensitivity between the two methods, and the larger linear, dynamic range of RNA-Seq [48,49]. Other possible causes of these differences may be that the SON samples for RNA-Seq were obtained by laser microdissection and the microarray SON samples were obtained by manual dissection of the SON, and the duration of SL was for 5 days in the RNA-seq study and for 7 days in the microarray study. A Venn diagram of intersections of the 552 genes from the RNA-Seq study (S9 Table) and the 1,309 genes from the microarray data (S13 Table) that changed with SL is shown in Fig 4. Of these genes, only 146 were found to intersect and the list of these genes and their fold-changes and ingenuity descriptions are presented in S15 Table. Since these 146 genes identified as definitely changing were confirmed by these two different methods, this represents a type of validation of the reliability of these conclusions. Also in the Venn diagram in Fig 4 there are 406 genes that are solely identified by the RNA-Seq method (S16 Table) and 1139 genes identified only by the microarray method (S17 Table). Many of the latter genes are substantially changed in expression by SL (e.g. with > twofold changes) and with statistically significant p-values, and in some cases have been validated by qPCR. In addition, many of the genes identified in this study as being expressed in the SON have also been validated by other independent methods, for example for Neuronatin(Peg5) and Pspss2 by differential hybridization [54], and for Trpv2, Vgf, and opsin by in situ hybridization or immunohistochemistry [29,55,56].

### Biological Significance of the SON Transcriptome Data

One can easily be overwhelmed by the abundance of data obtained from either the RNA-Seq or the microarray platforms. One clear value of these data is that it allows the investigator to determine whether the mRNA of interest is actually present in the tissue being studied (e.g., the SON), its relative abundance, and whether it responds to experimental perturbations (e.g., SL). One approach to organize such data is to analyze the complex transcriptomic and genomic information by using Ingenuity Pathway Analysis (IPA) (www.ingenuity.com). IPA analyzes data from expression profiling and proteomic experiments and generates large gene lists which suggest molecular interactions in the data and networks based on a large database in the literature. S18 Table shows a list of 25 networks predicted by IPA for the 552 RNA-Seq derived genes selected as being differentially expressed in the SON between SL and control rats. S6 Fig and Fig 5 illustrate the molecular interactions suggested for two of these networks.

The network shown in Fig 5 is of particular interest because it links the CREB family of genes which had been proposed as transcription factors that regulate transcription of the Avp gene in the Avp-MCNs through its CRE sites in its promoter that is activated by cyclic AMP [7,57,58]. It is known that the SON responds to hyperosmotic stimuli by increasing cAMP [39]. While the increase in Avp gene expression was initially thought to be caused by CREB activation, a study which injected a highly selective, dominant-negative CREB inhibitor (A-CREB) into the SON showed that this inhibitor which could block the induced expression of c-fos in response to acute hyperosmotic simulation, but did not inhibit the simultaneous increase in Avp gene transcription under the same circumstances [59]. In this regard, it is interesting that both the RNA-Seq and the microarray data identify three other CREB family members, Creb3l1, Atf4, and Atf5 [60], as being present in the SON. Each of these genes interact in the network shown in Fig 5, and all of them increase their expression in response to SL treatment (Table 3 and Table 8). None of these transcription factors’ actions would have been inhibited by the dominant-negative inhibitor, A-CREB [60,61], therefore all of these genes are candidates to bind the CRE sites in the Avp promoter and thereby regulate Avp gene expression. In this regard, a recent paper provides compelling evidence that one of these genes, CREB3L1, does indeed activate Avp gene expression by binding to the CRE-like and Gbox sequences between -170 and -120 bp in the Avp promoter [62]. This -170 to -120 bp region in the Avp promoter was also identified as a key domain involved in the cell-specific expression of the Avp gene in the Avp MCNs in in vivo experiments [63]. It would be interesting to determine if the other two CREB family candidates shown in Fig 5, Atf 4 and Atf5, also bind to these CRE-like and Gbox sequences in the Avp promoter and will also regulate the Avp gene’s expression in vivo, possibly in a synergistic manner. It is also notable that several other genes in the network in Fig 5, e.g., Cebpg, Psph and Tef also increase and some such as Dsp, and Agap2 decrease in expression during SL, but no member of the Akt gene family (a hub gene in this network) underwent a significant change in gene expression in response to the osmotic (SL) stimulus.

That there was also no change in expression of the ligand-dependent nuclear receptor RNAs in the SON in response to SL (Table 5) was surprising. This is because this family of transcription factors (nuclear receptors) have been implicated in the regulation of the Oxt gene, and have been hypothesized to act via a hormone responsive element found in the Oxt gene promoter [7,64] The latter data raise the question whether the expectation that a transcription factor gene that regulates a target gene must increase in expression when the target gene increases in expression under a specific physiological condition (such as for Oxt gene expression and SL). It is possible that such co-regulation need not be an absolute criterion for identifying a transcription factor candidate for any given target. Moreover, many of the genes in Table 5 that undergo substantial changes in gene expression in response to SL may be unrelated to the regulation of the Oxt and Avp genes in the SON, and are likely to be related to the other profound physiological and biochemical adaptive changes occurring in the MCNs during SL.

### Concluding Remarks

A fundamental question in molecular neuroscience is how specific neuronal phenotypes are formed and maintained in the central nervous system. It is generally recognized that the expression of specific genes during development and in the fully differentiated state are important determinents of phenotype. MCNs in the SON are highly specialized to release large amounts of Avp or Oxt into the blood stream and play critical roles in the regulation of body fluid homeostasis. The MCNs are osmosensory neurons and are excited by exposure to hypertonic solutions and inhibited by hypotonic solutions. In addition, the MCNs respond to systemic hypertonic and hypotonic stimulation with large changes in the expression of their Avp and Oxt genes, and microarray studies have shown that these osmotic perturbations also cause large changes in global gene expression in the HNS. In this paper, we examine gene expression in the rat SON under normosmotic (control) and chronic salt-loading SL) conditions by the first time using “new-generation”, RNA sequencing (RNA-Seq) methods. We reliably detect 9,709 genes as being present in the SON by RNA-Seq. We also compare the SON transcriptome resolved by RNA-Seq methods with the SON transcriptome determined by Affymetrix microarray methods in rats under the same osmotic conditions. We find that there are 6,466 genes present in the SON that are represented in both data sets, although 1,040 of the expressed genes were found only in the microarray data, and 2,762 of the expressed genes are selectively found in the RNA-Seq data and not the microarray data. We find 552 of these genes were changed in expression as a result of chronic SL. These genes reflect diverse functions, and 42 of these are involved in either transcriptional or translational processes. These data provide the research community a comprehensive view of the transcriptome in the SON under normosmotic conditions and the changes that occur in specific gene expression evoked by salt loading. The RNA-Seq database we present in this paper is a valuable resource for the further study of SON functions during osmoregulation, and should drive new research by identifying new molecular targets to be interrogated with regard to osmotic regulation in the SON. In this regard, our IPA analysis suggests that a key network involving several Creb family transcription factors, e.g, Creb3l1, Atf4, and Atf5 (see Fig 5) should be worthy of further study under SL conditions. This RNA-Seq database also provides a baseline for the determination of the molecular differences between the Oxt and Avp phenotypes. As noted above, the SON is heterogeneous with respect to its cellular phenotypes. Hence, it is not possible from these RNA-Seq data of the whole SON to computationally deconvolute the individual contributions of the Oxt and Avp MCNs. However, transgenic rats containing green fluorescent (eGFP) Avp MCNs [65] or red fluorescent (mRFP1) Oxt MCNs [66] now exist and methods have been developed to dissect single, fluorescent neurons [28,67] for high throughput RNA analyses [68,69,70]. Therefore, it should now be possible to do RNA-Seq studies of the specific Oxt and Avp MCN phenotypes in the SON and to identify the unique RNAs that are present and are changed by SL in the transcriptomes of each of these individual phenotypes.

## Supporting Information

S1 Fig Micrograph illustrating magnocellular neurons (MCNs) in a salt loaded SON mounted on a PEN membrane frame slide and visualized by a Arcturus XT laser capture microscope. (EPS)

S2 Fig RNA-Seq Analysis Workflow. (EPS)

S3 Fig Micrograph of a section of the rat SON stained immunochemically red using a pan-specific antibody against rat neurophysin (a marker of all MCNs) and histochemically counterstained blue by Dapi, an nuclear marker. (EPS)

S4 Fig Microarray Analysis Workflow. (EPS)

S5 Fig Noise analysis comparison between A) RNA-seq and B) Microarray. (A) XY scatter plot of the RNA-Seq Mean Expression (x-axis) vs the observed Coefficient of Variation (y-axis) for 21,083 genes.
(EPS)

S6 Fig Network Analysis depicting gene products and known relationships between them for the top-ranked scoring network by Ingenuity (http://www.ingenuity.com/) when provided list of differentially expressed genes observed between salt-loaded and control by RNA-Seq (based on data in S9 Table). (PDF)

S7 Fig qPCR determinations of fold changes in gene expression in the SON in response to SL. (PDF)

S8 Fig Comparison of relative expression for Agrn transcripts between SL and Control. (EPS)

S9 Fig Comparison of relative expression for Nnat transcripts between SL and Control. (EPS)

S1 Table Measurements of MCN and non-MCN cells in the SON. (DOCX)

S2 Table Total RNA obtained from Normal and Salt Loaded Rat SONs by LCM. (DOCX)

S3 Table List of primer sequences used for qPCR validation of gene expression in the supraoptic nucleus of either euhydrated or 5-day salt-loaded rats. (DOC)

S4 Table 9, 321 RNA-seq genes present in Con SON. (XLS)

S5 Table 9,315 RNA-seq genes present in SL SON. (XLS)

S6 Table 9,709 RNA-seq genes present in Con and/or SL SON. (XLS)

S7 Table Statistical Testing Results for the 9,709 RNA-Seq genes present in Con and/or SL SON. (XLS)

S8 Table Statistical Testing Results for 772 specific Transcription and Translation Regulators detected in SON by RNA-seq. (XLS)

S9 Table 552 Genes selected from RNA-seq Results using (corrected P < 0.10, FCM > = 1.5 criteria). (XLS)

S10 Table 10,382 Microarray gene probes present in Con SON. (XLS)

S11 Table 10,476 Microarray gene probes present in SL SON. (XLS)

S12 Table 11,293 Microarray gene probes present in Con and/or SL SON. (XLS)

S13 Table 1,309 Microarray gene probes selected as differentially expressed between SL and Control. (XLS)

S14 Table 171 Microarray gene probes representing 146 unique genes selected as differentially expressed between SL and Control which are found by both Microarray and RNA-Seq analyses (see S15 Table). (XLS)

S15 Table Genomic locations for the 146 unique genes selected as differentially expressed between SL and Control which are found by both RNA-Seq and Microarray. (XLS)

S16 Table The 406 unique genes selected as differentially expressed between SL and Control found by RNA-Seq and not by microarray analysis. (XLS)

S17 Table The 1163 unique genes selected as differentially expressed between SL and Control found by microarray and not by RNA-seq analysis. (XLS)

S18 Table Top 25 Networks for the 552 RNA-Seq genes selected as differentially expressed between SL and Control. (XLS)

S19 Table Enriched Functions for the 552 RNA-Seq genes selected as differentially expressed between SL and Control. (XLS)

S20 Table Enriched Pathways for the 552 RNA-Seq genes selected as differentially expressed between SL and Control. (XLS)

S21 Table Expression of alternatively spliced transcripts of Agrn and Nnat in Control and SL SONs.* (EPS)
