# Correction: A RNA-Seq Analysis of the Rat Supraoptic Nucleus Transcriptome: Effects of Salt Loading on Gene Expression

**DOI:** 10.1371/journal.pone.0131892

**Published:** 2015-06-25

**Authors:** Kory R. Johnson, C. C. T. Hindmarch, Yasmmyn D. Salinas, YiJun Shi, Michael Greenwood, See Ziau Hoe, David Murphy, Harold Gainer

There are a number of errors in the captions for S1 to S9 Fig. in the Supporting Information. Please see the complete, correct S1 to S9 Fig. captions here.

**S1 Fig. Micrograph illustrating magnocellular neurons (MCNs) in a salt loaded SON mounted on a PEN membrane frame slide and visualized by a Arcturus XT laser capture microscope**. Large blood vessel adjacent to the SON is shown in the upper left corner. Scale line is 100um.

**S2 Fig. RNA-Seq Analysis Workflow.** (A) Overview of analysis steps performed. Step 1, "Sequence Adaptor Clipping”. Read pairs, 101bp in length, were adaptor clipped via FASTQ/A Clipper (http://hannonlab.cshl.edu). Broken read pairs as a result of clipping were discarded. Step 2, “Sequence Quality Inspection”. Intact read pairs remaining post-clipping were import into CLCbio and the “Create Sequencing QC Report” tool used to generate one report per sample. These reports, each containing per-sequence and per-base quality statistics, were individually inspected and cross-compared to define universal hard-trimming rules to be applied to all samples. Step 3, “Sequence Quality Trimming & Filtering”. The CLCbio “Trim Sequences” tool was used to hard-remove the first 15nt from the 5’ end and the last 1nt from the 3’ end of each read pair. The tool was also used to dynamically-trim away nucleotides having a call accuracy rate less than 95%. Read pairs having at least one sequence containing more than two ambiguities were also discarded as part of this Step as were read pairs having at least one sequence with a post trimmed length less than 15 nucleotides. Step 4, “Sequence Alignment and Enumeration”. The CLCbio “RNA-Seq Analysis” tool was used to align read pairs to the Rat Genome (RN5) by sample using default parameters. Output provided by the tool included a Reads per kilo base per million (RPKM) expression value for 26,313 genes. Step 5, “RPKM Expression Pedelstalling”. Output from Step 4 was imported into R (http://www.r-project.org/) and a value of two added to each RPKM expression value per sample. Step 6, “RPKM Expression Transformation”. Pedestalled values from Step 5 (RPKM+2) were Log2 transformed using standard commands in R then filtered to keep only those genes having a post-transformed expression value (Log2(RPKM+2)) >1 for at least one sample. Step 7, “RPKM Expression Normalization”. Transformed values from Step 5 were quantile normalized using standard commands in R. Step 8, “Exploratory Analysis”. Normalized values from Step 7 (Quantile(Log2(RPKM+2))) were interrogated in R by Tukey box plot, covariance-based principal component analysis (PCA) scatter plot and Pearson correlation-based heat map to confirm absence of outliers. Step 9, “Noise Analysis”. For each gene, the coefficient of variation (CV) and mean expression was calculated by sample class using standard commands in R then modeled by sample class using the lowess() command. Step 10, “Confidence Criterion Selection”. Lowess fits from Step 9 were visually inspected to define the mean expression value across sample classes at which the linear relationship between CV (i.e., noise) and mean expression (i.e., signal) is grossly lost. Step 11, “Gene Filtering and Flooring”. Genes with an expression value less than the value defined in Step 10 were floored to equal that value using standard commands in R. Genes not having at least one sample in either class with an expression value greater than the floored-to-value were discarded prior to Step 7 as noise-biased. Step 12, "Statistical Testing". Expression for genes remaining post Step 11 were compared between classes via Welch-modified t-test in R under Benjamini and Hochberg (BH) False Discovery Rate (FDR) Multiple Comparison Correction (MCC) condition. Step 13, "Gene Selection". Test results from Step 12 were used to select and subset only those genes having both a corrected P < 0.05 and an absolute difference of mean expression between classes > = 1.50. Step 14, “Confirmatory Analysis”. Expression values for genes subset as part of Step 13 were interrogated in R by covariance-based principal component analysis (PCA) scatter plot and Pearson correlation-based heat map to confirm sample grouping by class. Step 15, “Pathway Enrichment”. Genes subset as part of Step 13 were import into IPA (www.ingenuity) and enriched biological functions identified. Step 16, “Function Enrichment”. Genes subset as part of Step 13 were import into IPA (www.ingenuity) and enriched biological functions identified. (B) Tukey Box Plot. Plot was generated as part of analysis Step 8, "Exploratory Analysis". Plot depicts 6 samples along the x-axis (3 controls, green-filled; 3 salt-loaded, red-filled). Plot was generated in R using 26,313 gene expression values (Quantile(Log2(RPKM+2))) per sample (y-axis). Plot shows no remarkable differences per expression distribution location, spread and skew. (C) covariance-based Principal Component Analysis scatter plot. Plot was generated as part of analysis Step 8, "Exploratory Analysis". Plot depicts 6 samples (3 controls, green-filled; 3 salt-loaded, red-filled). Plot was generated in R using 26,313 gene expression values (Quantile(Log2(RPKM+2))) per sample. Plot shows no remarkable outliers. (D) Pearson correlation heat map. Map was generated as part of analysis Step 8, "Exploratory Analysis". Map depicts 6 samples along the diagonal (3 control, green-outlined; 3 salt-loaded, red-outlined). Map was generated in R using 26,313 gene expression values (RMA) per sample. Map shows no remarkable outliers. (E) XY scatter plot. Plot was generated as part of analysis Step 9, "Noise Analysis". Plot depicts the relationship between mean expression (x-axis) and the coefficient of variation of expression (y-axis) by sample class (control, green line; salt-loaded, red line). Plot was generated in R using 26,313 gene expression values (Quantile(Log2(RPKM+2))) per sample. Dashed vertical black-colored line occurring along the x-axis at value 3.0 depicts the “Confidence Criterion” selected as part of analysis Step 10. (F) Volcano plot. Plot was generated as part of analysis Step 13, "Gene Selection”. Plot depicts the linear fold changes (x-axis) observed between salt-loaded and control mean expression for 9,709 genes verses the significance for those changes (y-axis). The vertical dashed black-colored lines in the plot represent mean fold change magnitude (FCM) = 1.50. The horizontal dashed black-colored line represents corrected P = 0.05. Gray-colored circles in the plot (n = 9,430) represent those genes with FCM < 1.50 and/or corrected P > = 0.05. Black-colored downward-pointing triangles in the plot (n = 138) represent those genes with a FC < = -1.50 and corrected P < 0.05. Black-colored upward-pointing triangles in the plot (n = 141) represent those genes with a FC > = 1.50 and corrected P < 0.05. (G) covariance-based Principal Component Analysis scatter plot. Plot was generated as part of analysis Step 14, "Confirmatory Analysis". Plot depicts 6 samples (3 controls, green-filled; 3 salt-loaded, red-filled). Plot was generated in R using expression (Quantile(Log2(RPKM+2))) for the 279 genes selected as part of analysis Step 13. Plot confirms excellent grouping by class using the genes selected. (H) Pearson correlation heat map. Map was generated as part of analysis Step 14, "Confirmatory Analysis". Map depicts 6 samples along the diagonal (3 control, green-outlined; 3 salt-loaded, red-outlined). Map was generated in R using expression (Quantile(Log2(RPKM+2))) for the 279 genes selected as part of analysis Step 13. Map confirms excellent grouping by class using the genes selected.

**S3 Fig. Micrograph of a section of the rat SON stained immunochemically red using a pan-specific antibody against rat neurophysin (a marker of all MCNs) and histochemically counterstained blue by Dapi, a nuclear marker.** The dotted line circumscribes the outside border of the SON. Note that the MCN nuclei are large and pale blue while the non-neuronal cells (e.g, glia) are small and intensely stained blue. This distinction permitted the determination of the MCN and non-neuronal cell numbers shown in Table S1. The scale line is 100um.

**S4 Fig. Microarray Analysis Workflow.** A) Overview of analysis steps performed. Step 1, ".CEL File Summarization & Normalization”. CEL files were imported into the Affymetrix Expression Console (http://affymetrix.com) and RMA-based expression generated for 31,099 gene probes per sample. Step 2, “Batch Correction via Baseline Subtraction”. Sample expression generated as part of Step 1 included use of CEL files as input that were generated from two separate batches with one common sample run between them. To eliminate batch-to-batch differences, baseline subtraction was performed between batches using the common sample. Step 3, “Exploratory Analysis”. Expression values from Step 2 were interrogated in R (http://www.r-project.org/) by Tukey box plot, covariance-based principal component analysis (PCA) scatter plot and Pearson correlation-based heat map to confirm absence of outliers. Step 4, “Noise Analysis”. For each gene probe, the coefficient of variation (CV) and mean expression was calculated by sample class using standard commands in R then modeled by sample class using the lowess() command. Step 5, “Confidence Criterion Selection”. Lowess fits from Step 4 were visually inspected to define the mean expression value across sample classes at which the linear relationship between CV (i.e., noise) and mean expression (i.e., signal) is grossly lost. Step 6, “Gene Filtering and Flooring”. Gene probes with an expression value less than the value defined in Step 5 were floored to equal that value using standard commands in R. Gene probes not having at least one sample in either class with an expression value greater than the floored-to-value were discarded prior to Step 7 as noise-biased. Step 7, "Statistical Testing". Expression for gene probes remaining post Step 6 were compared between classes via Welch-modified t-test in R under Benjamini and Hochberg (BH) False Discovery Rate (FDR) Multiple Comparison Correction (MCC) condition. Step 8, "Gene Selection". Test results from Step 7 were used to select and subset only those gene probes having both a corrected P < 0.05 and an absolute difference of mean expression between classes > = 1.50. Step 9, “Confirmatory Analysis”. Expression values for gene probes subset as part of Step 8 were interrogated in R by covariance-based principal component analysis (PCA) scatter plot and Pearson correlation-based heat map to confirm sample grouping by class. Step 10, “Probe to Gene Annotation”. Gene probes subset as part of Step 8 were imported into IPA (www.ingenuity) and known gene annotations assigned. Step 11, “Pathway Enrichment”. Gene probes having known gene annotation were import into IPA (www.ingenuity) and enriched biological functions identified. Step 12, “Function Enrichment”. Gene probes having known gene annotation were import into IPA (www.ingenuity) and enriched biological functions identified. (B) Tukey Box Plot. Plot was generated as part of analysis Step 3, "Exploratory Analysis". Plot depicts 10 samples along the x-axis (5 controls, green-filled; 5 salt-loaded, red-filled). Plot was generated in R using 31,099 gene probe expression values (RMA) per sample (y-axis). Plot shows no remarkable differences per expression distribution location, spread and skew. (C) covariance-based Principal Component Analysis scatter plot. Plot was generated as part of analysis Step 3, "Exploratory Analysis". Plot depicts 10 samples (5 controls, green-filled; 5 salt-loaded, red-filled). Plot was generated in R using 31,099 gene probe expression values (RMA) per sample. Plot shows no remarkable outliers. (D) Pearson correlation heat map. Map was generated as part of analysis Step 3, "Exploratory Analysis". Map depicts 10 samples along the diagonal (5 control, green-outlined; 5 salt-loaded, red-outlined). Map was generated in R using 31,099 gene probe expression values (RMA) per sample. Map shows no remarkable outliers. (E) XY scatter plot. Plot was generated as part of analysis Step 4, "Noise Analysis". Plot depicts the relationship between mean expression (x-axis) and the coefficient of variation of expression (y-axis) by sample class (control, green line; salt-loaded, red line). Plot was generated in R using 31,099 gene probe expression values (RMA) per sample. Dashed vertical black-colored line occurring along the x-axis at value 6.5 depicts the “Confidence Criterion” selected as part of analysis Step 5. (F) Volcano plot. Plot was generated as part of analysis Step 8, "Gene Selection”. Plot depicts the linear fold changes (x-axis) observed between salt-loaded and control mean expression for 11,293 gene probes verses the significance for those changes (y-axis). The vertical dashed black-colored lines in the plot represent mean fold change magnitude (FCM) = 1.50. The horizontal dashed black-colored line represents corrected P = 0.05. Gray-colored circles in the plot (n = 9,984) represent those gene probes with FCM < 1.50 and/or corrected P > = 0.05. Black-colored downward-pointing triangles in the plot (n = 417) represent those gene probes with a FC < = -1.50 and corrected P < 0.05. Black-colored upward-pointing triangles in the plot (n = 892) represent those gene probes with a FC > = 1.50 and corrected P < 0.05. (G) covariance-based Principal Component Analysis scatter plot. Plot was generated as part of analysis Step 9, "Confirmatory Analysis". Plot depicts 10 samples (5 controls, green-filled; 5 salt-loaded, red-filled). Plot was generated in R using expression (RMA) for the 1,309 gene probes selected as part of analysis Step 8. Plot confirms excellent grouping by class using the gene probes selected. (H) Pearson correlation heat map. Map was generated as part of analysis Step 9, "Confirmatory Analysis". Map depicts 10 samples along the diagonal (5 control, green-outlined; 5 salt-loaded, red-outlined). Map was generated in R using expression (RMA) for the 1,309 gene probes selected as part of analysis Step 8. Map confirms excellent grouping by class using the gene probes selected.

**S5 Fig. Noise analysis comparison between A) RNA-seq and B) Microarray.** (A) XY scatter plot of the RNA-Seq Mean Expression (x-axis) vs the observed Coefficient of Variation (y-axis) for 21,083 genes. Gray-colored circles represent the genes with two circles used per gene, one to represent the RNA-Seq mean expression and coefficient of variation (C.V.) for salt-loaded (SL) samples and the other to represent the RNA-Seq mean expression and C.V. for control samples. The red-colored curve in the plot depicts the locally weighted scatter plot smoothing fit (y-axis~x-axis) of the RNA-Seq mean expression and C.V. across all genes for the SL samples. The green-colored line in the plot depicts the locally weighted scatter plot smoothing fit (y-axis~x-axis) of the RNA-Seq mean expression and C.V. across all genes for the control samples. The Black vertical dashed line represents the RNA-Seq mean expression value selected as the noise threshold for the data (value = 3). Sample-level expression values less than the noise selected threshold were floored to equal this value if less. While, genes not having at least one sample with an expression value greater than the noise selected threshold were discarded as non-informative prior to statistical testing (see Figure S2, Step 11, “Gene Filtering and Flooring”). (B) XY scatter plot of the Microarray Mean Expression (x-axis) vs the observed Coefficient of Variation (y-axis) for 31,099 gene probes. Gray-colored circles represent the gene probes with two circles used per probe, one to represent the Microarray mean expression and coefficient of variation (C.V.) for salt-loaded (SL) samples and the other to represent the Microarray mean expression and C.V. for control samples. The red-colored curve in the plot depicts the locally weighted scatter plot smoothing fit (y-axis~x-axis) of the Microarray mean expression and C.V. across all genes for the SL samples. The green-colored line in the plot depicts the locally weighted scatter plot smoothing fit (y-axis~x-axis) of the Microarray mean expression and C.V. across all genes for the control samples. The Black vertical dashed line represents the Microarray mean expression value selected as the noise threshold for the data (value = 6.5). Sample-level expression values less than the noise selected threshold were floored to equal this value if less. While, genes not having at least one sample with an expression value greater than the noise selected threshold were discarded as non-informative prior to statistical testing (see Figure S4, Step 6, “Gene Filtering and Flooring”).

**S6 Fig. Network Analysis depicting gene products and known relationships between them for the top-ranked scoring network by Ingenuity (http://www.ingenuity.com/) when provided list of differentially expressed genes observed between salt-loaded and control by RNA-Seq (based on data in Table S9).** Gene products are represented using circle-shaped symbols with connected edges drawn between them to describe interactions (solid edge = direct interaction, dashed edge = indirect interaction). Color-filled shapes indicate the direction of differential expression observed between salt-loaded and control (green = up, red = down). Circle-shaped symbols not color-filled represent gene products not observed differentially expressed between salt-loaded and control.

**S7 Fig. qPCR determinations of fold changes in gene expression in the SON in response to SL.** Ordinate shows qRT-PCR values for SONs from control rats (CT) and SONs from rats that were salt loaded for 5 days (SL5).

**S8 Fig. Comparison of relative expression for Agrn transcripts between SL and Control.** Figure describes the relative expression occurring between Agrn transcripts ENSRNOT00000045678 and ENSRNOT00000046315 by RNA-seq across 3 Control (C) and 3 salt-loaded (SL) samples. Only the last 4 exons occurring in the Agrn gene on chr 5 (hg19) are depicted. Star-shaped symbols found in the Figure denote the exon between the two transcripts that is variable. Statistical comparison of the expression between sample classes by transcript is reported in Table S21. Results suggest both transcripts are expressed; with the mean expression occurring lower for both transcripts under salt-loaded condition.

**S9 Fig. Comparison of relative expression for Nnat transcripts between SL and Control.** Figure describes the relative expression occurring between Nnat transcripts ENSRNOT00000072502 and ENSRNOT00000034166 by RNA-seq across 3 Control (C) and 3 salt-loaded (SL) samples. All exons occurring in the Nnat gene on chr 3 (hg19) are depicted. Star-shaped symbols found in the Figure denote the exon between the two transcripts that is variable. Statistical comparison of the expression between sample classes by transcript is reported in Table X. Results suggest both transcripts are expressed; with the mean expression occurring lower for both transcripts under salt-loaded condition.
