# Supplementary figures and images for: A RNA-Seq Analysis of the Rat Supraoptic Nucleus Transcriptome: Effects of Salt Loading on Gene Expression
Source: PLoS One. 2015 Apr 21;10(4):e0124523. doi: 10.1371/journal.pone.0124523 (PMC4405539; doi:10.1371/journal.pone.0124523)

Fig. S6

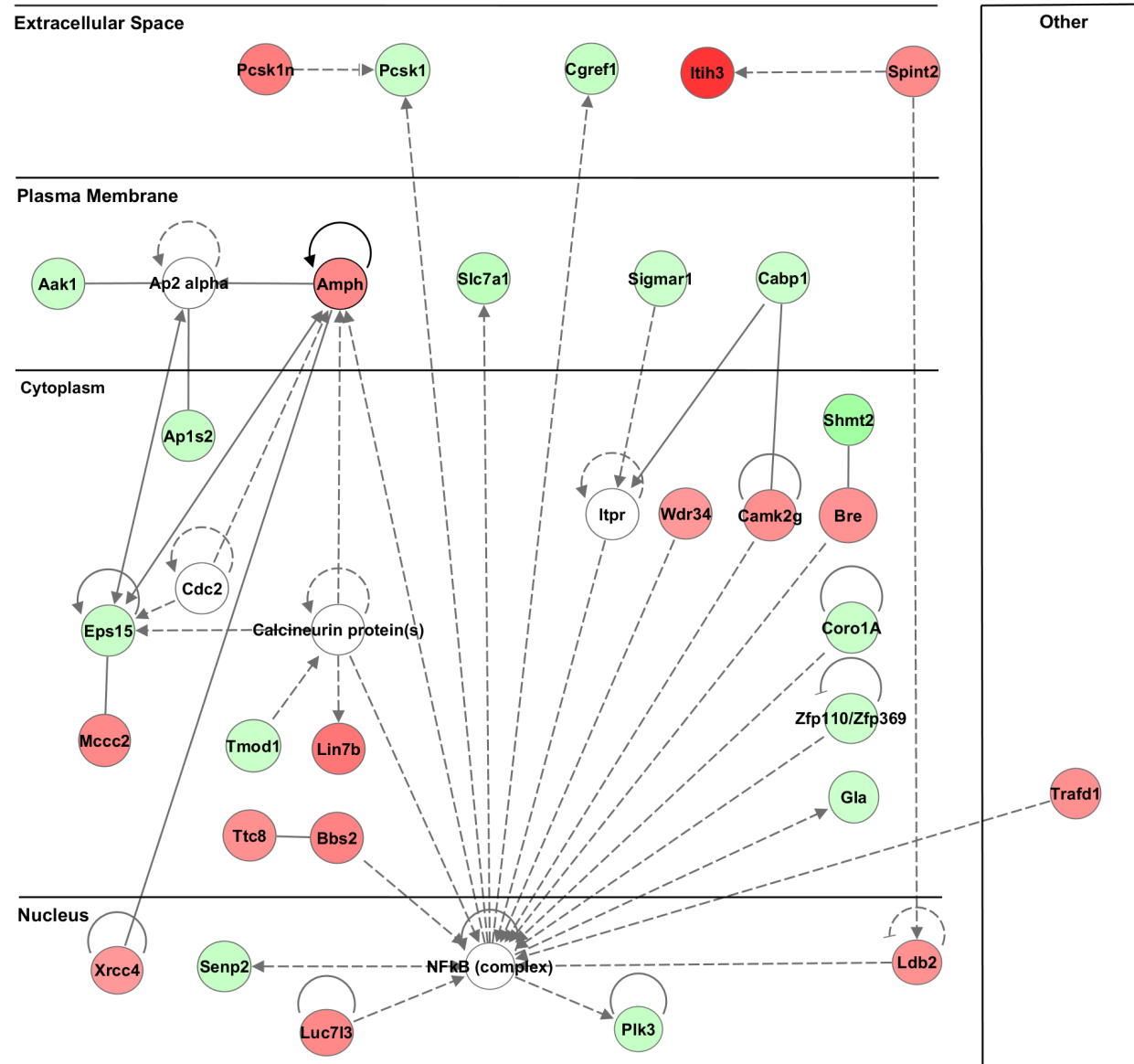

Supplement: S6 Fig — (PDF) [file pone.0124523.s006.pdf]
