# Supplementary material for: A RNA-Seq Analysis of the Rat Supraoptic Nucleus Transcriptome: Effects of Salt Loading on Gene Expression
Source: PLoS One. 2015 Apr 21;10(4):e0124523. doi: 10.1371/journal.pone.0124523 (PMC4405539; doi:10.1371/journal.pone.0124523)
Supplement: S7 Fig — (PDF) [file pone.0124523.s007.pdf]

Fig. S7

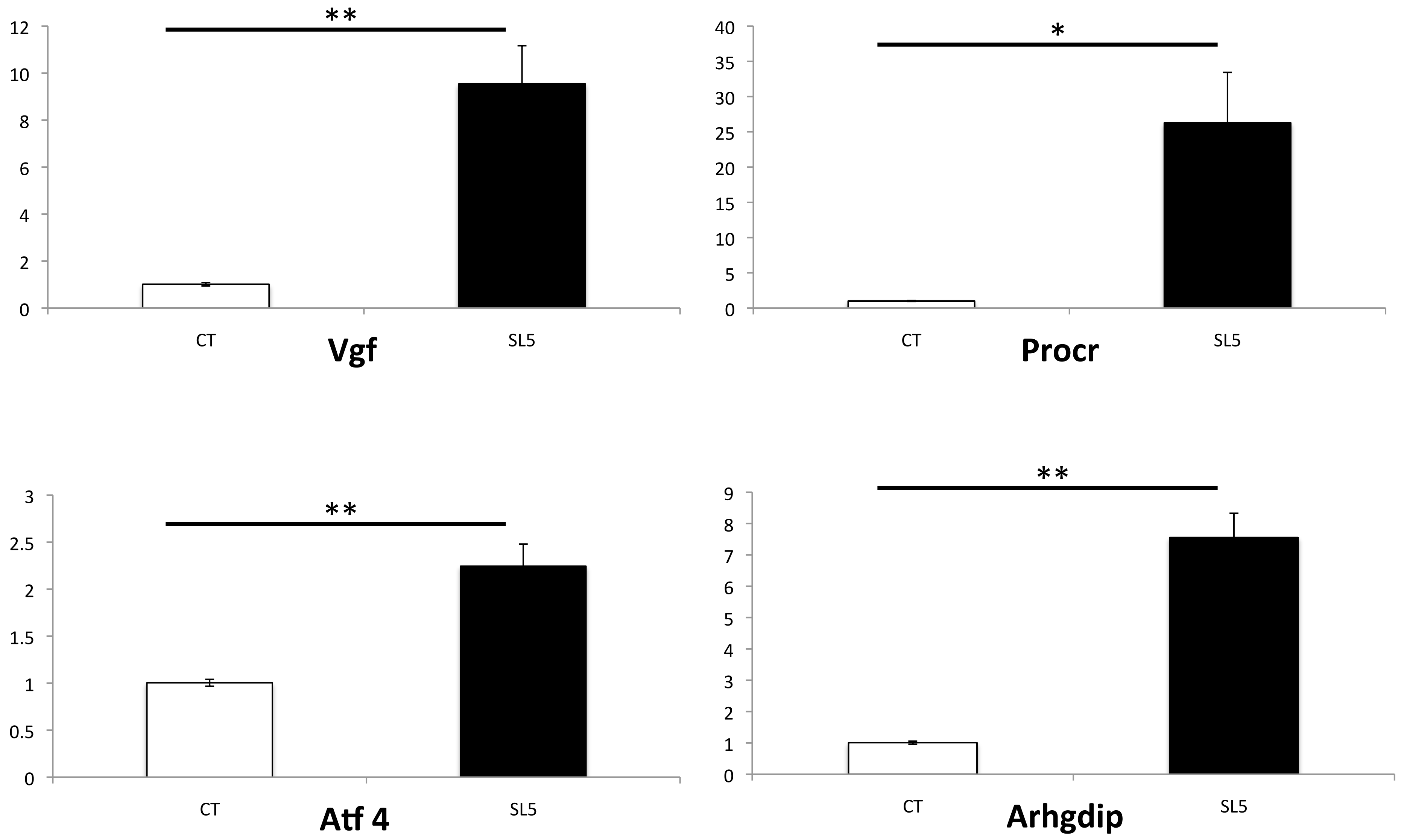

t-test * = <0.05; ** = <0.01; NS = not significant

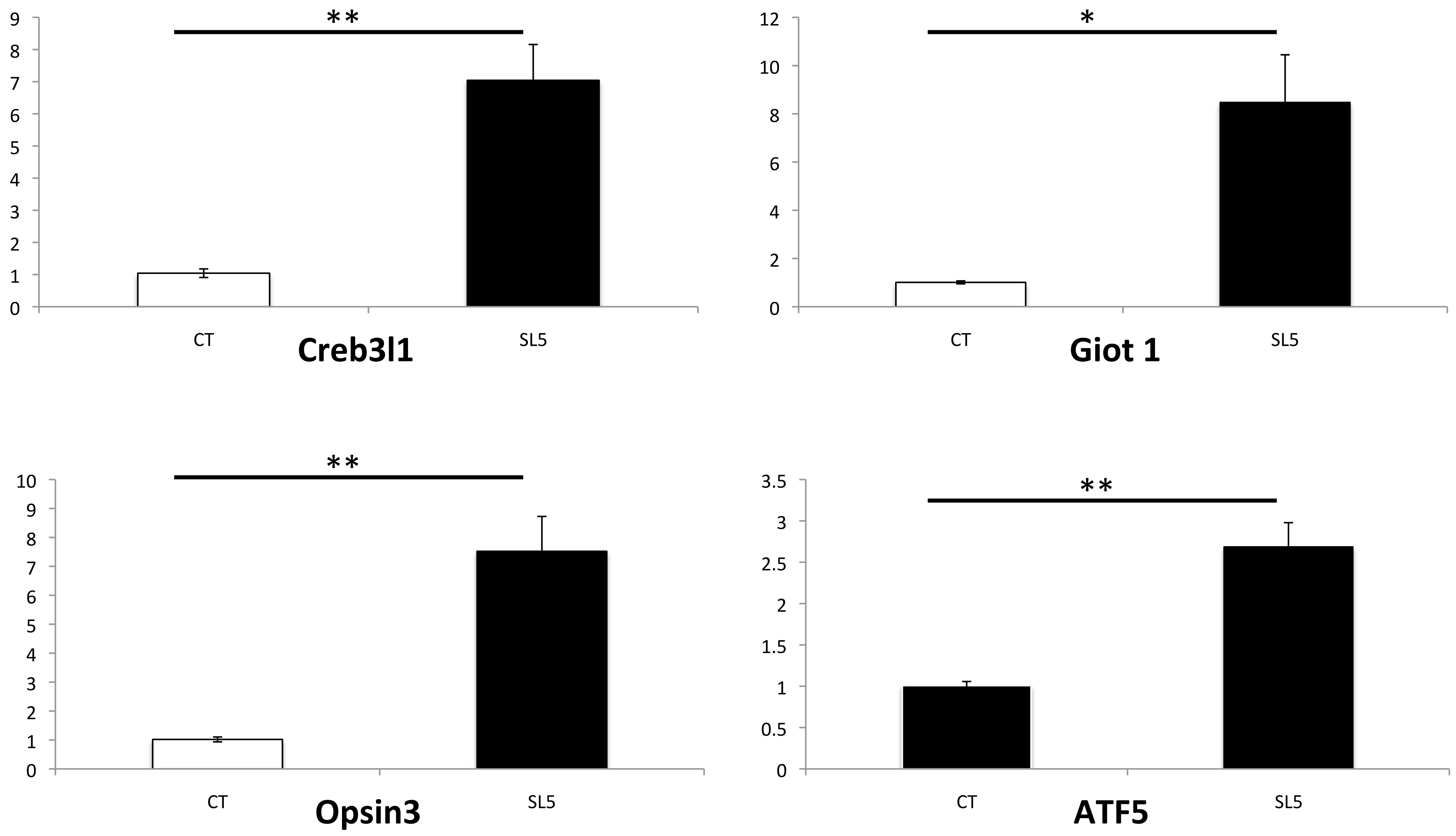

t-test * = <0.05; ** = <0.01; NS = not significant

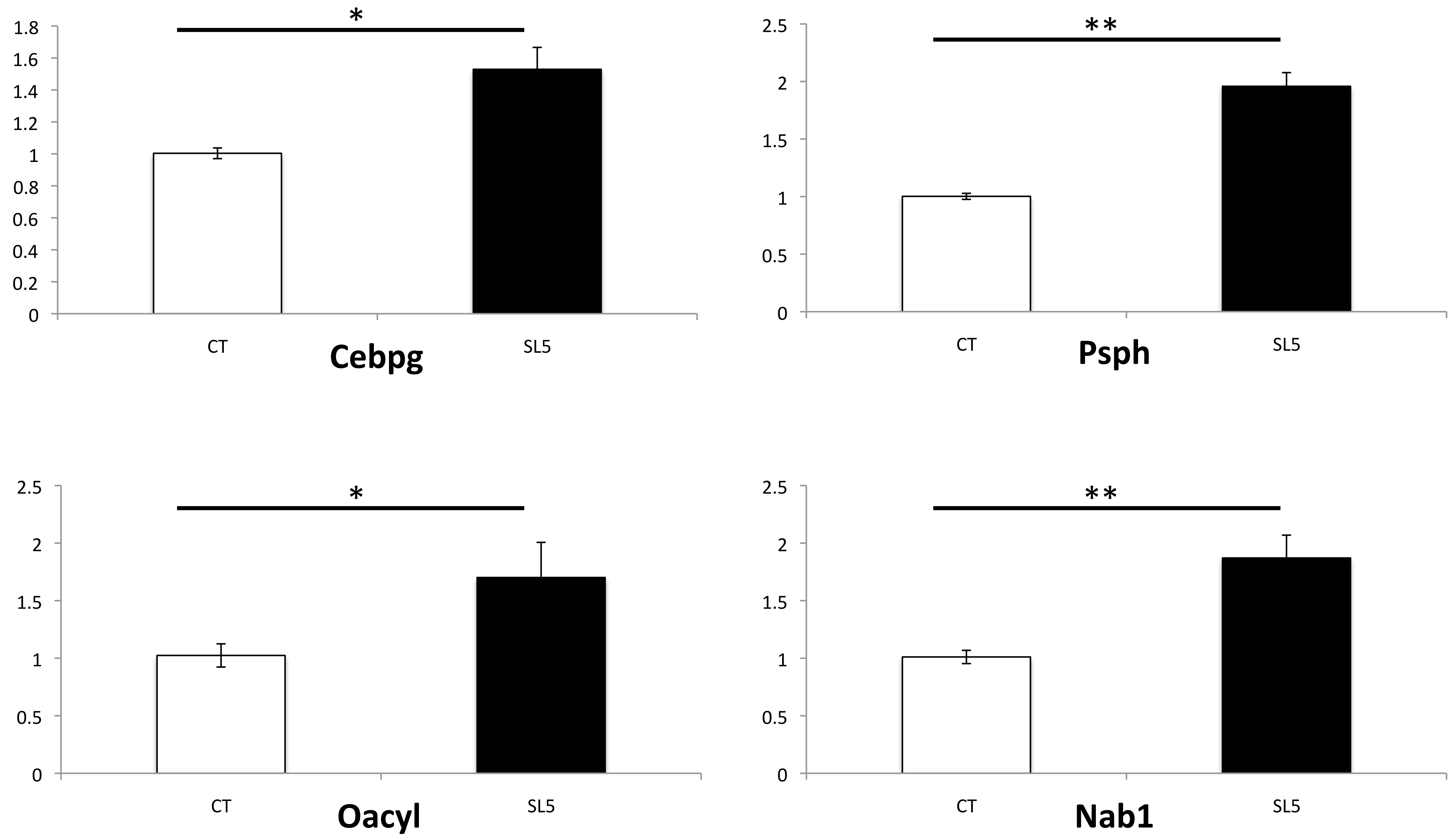

t-test * = <0.05; ** = <0.01; NS = not significant

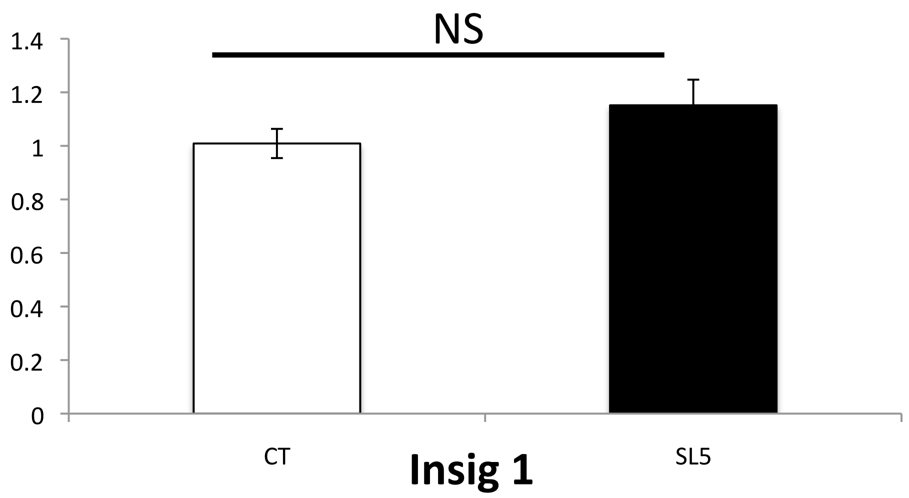

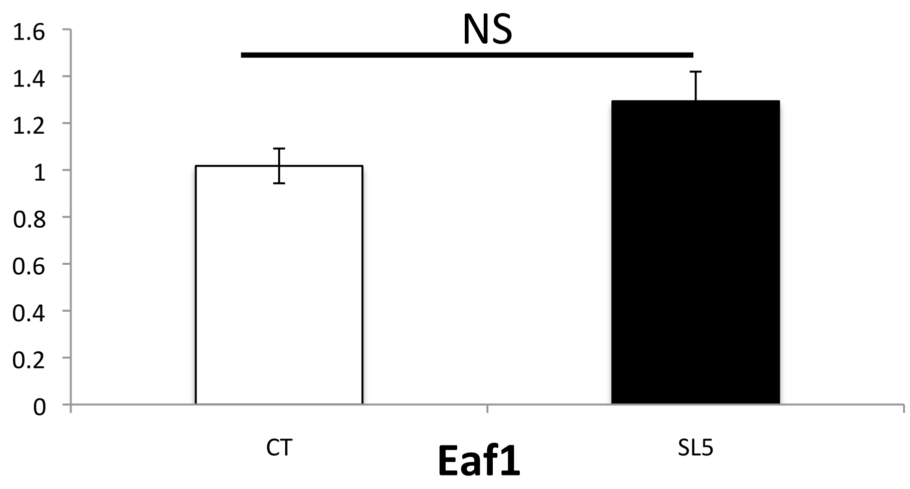

t-test * = <0.05; ** = <0.01; NS = not significant

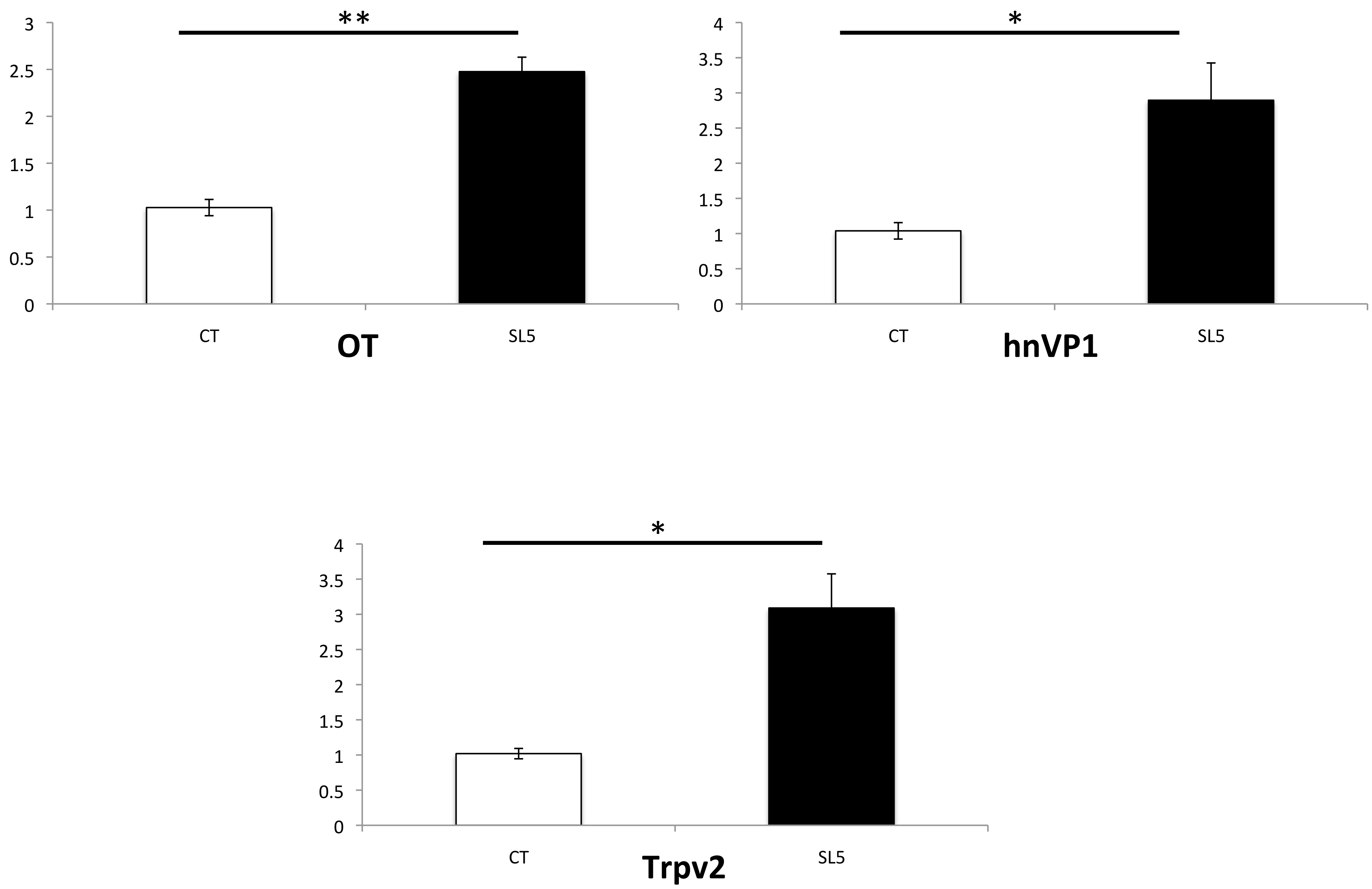

t-test * = <0.05; ** = <0.01; NS = not significant
