# Supplementary material for: A RNA-Seq Analysis of the Rat Supraoptic Nucleus Transcriptome: Effects of Salt Loading on Gene Expression
Source: PLoS One. 2015 Apr 21;10(4):e0124523. doi: 10.1371/journal.pone.0124523 (PMC4405539; doi:10.1371/journal.pone.0124523)
Supplement: S1 Table — (DOCX) [file pone.0124523.s010.docx]

Table S1

Measurements of MCN and non-MCN cells in the SON

Number of Cells

Section MCNs non-MCNs ratio

1 96 181 1.9

2 102 173 1.7

3 77 147 1.9

4 79 173 2.2

5 88 186 2.1

---------------------------------------------------------------------------------------

Ave ratio = 1.96 non-MCNs/ MCNs

Dapi stained (MCN and non MCN) nuclei and PS 45 stained MCNs

Measured from Salt-Loaded rat SONs
