# Supplementary material for: A RNA-Seq Analysis of the Rat Supraoptic Nucleus Transcriptome: Effects of Salt Loading on Gene Expression
Source: PLoS One. 2015 Apr 21;10(4):e0124523. doi: 10.1371/journal.pone.0124523 (PMC4405539; doi:10.1371/journal.pone.0124523)
Supplement: S2 Table — (DOCX) [file pone.0124523.s011.docx]

Table S2

Total RNA obtained from Normal and Salt Loaded

Rat SONs by LCM (ng/ SON pair)

Normal Salt Loaded

260 658

344 552

274 748

260 963

386 523

308 ----

Ave (SD) 305± (52) 689± (177)

(SE) (21) (79)

Ratio of SL/N = 2.3
