# Supplementary material for: A RNA-Seq Analysis of the Rat Supraoptic Nucleus Transcriptome: Effects of Salt Loading on Gene Expression
Source: PLoS One. 2015 Apr 21;10(4):e0124523. doi: 10.1371/journal.pone.0124523 (PMC4405539; doi:10.1371/journal.pone.0124523)
Supplement: S3 Table — (DOC) [file pone.0124523.s012.doc]

**Table S3: List of primer sequences used for qPCR validation of gene expression in the supraoptic nucleus of either euhydrated or 5-day salt-loaded rats.**

| **Primer** | **Forward** | **Reverse** |
| --- | --- | --- |
| Rpl19 | GCGTCTGCAGCCATGAGTA | TGGCATTGGCGATTTCGTTG |
| Atf5 | CAGTGCCTAGGGTACAGGAG | AATGGAGGGACAGGGTGAAG |
| Cebpg | CAGCTCAAGGAGGAGAACGA | CCGTAGTTTCCGTGCTGATG |
| Psph | TGGCCAAATTCTGTGGTGTG | AGCGCATCTTTGAAAGGCAA |
| Trpv2 | GAGTCACCATTCCAGAGGGA | GTTCAGCACAGCCTTCATCA |
| Nab1 | TACAGCATGTCAGGGGACAG | GGTAGAGCCTCTGGGTTCAA |
| Oacyl | TGACGGCCTTCACTTTGCTA | AGCGTATTCTTTGGGCTTCAG |
| Insig1 | CACGTCCCCAGATTTCCTCT | GGCTTTTCTGGAACACCCAT |
| Procr | AACGACGTGGTCTTTCCTCT | TATGGCAGTCTTTGGCTGGA |
| Vgf | ATGAGTTGCCGGACTGGAA | CGCGGCCGAATGTAGTTTG |
| Eaf1 | ACAGGCGTTGGGGATAGAAT | GCAGTGCCATGGAGAGAGAA |
| Creb3l1 | GAGACCTGGCCAGAGGATAC | GTCAGTGAGCAAGAGAACGC |
| Giot1 | GACACTTCCGGTCCGTCATAG | GCCTCACTCAAGCACCCAGT |
| Opsin3 | ATGGCTATGGACACCTGGTC | CAGAGGAGTTGCAGAAGGGA |
| Oxt | TGAGCAGGAGGGGGCCTAGC | TGCAAGAGAAATGGGTCAGTGGC |
| hnAvp | GAGGCAAGAGGGCCACATC | CTCTCCTAGCCCATGACCCTT |
| Atf4 | Quantitect Assay (Qiagen) | |
| Arhgdip | Quantitect Assay (Qiagen) | |
